# An integrated transcriptomic cell atlas of human endoderm-derived organoids

**DOI:** 10.1038/s41588-025-02182-6

**Published:** 2025-05-12

**Authors:** Quan Xu, Lennard Halle, Soroor Hediyeh-zadeh, Merel Kuijs, Rya Riedweg, Umut Kilik, Timothy Recaldin, Qianhui Yu, Isabell Rall, Tristan Frum, Lukas Adam, Shrey Parikh, Raphael Kfuri-Rubens, Manuel Gander, Dominik Klein, Fabiola Curion, Zhisong He, Jonas Simon Fleck, Koen Oost, Maurice Kahnwald, Silvia Barbiero, Olga Mitrofanova, Grzegorz Jerzy Maciag, Kim B. Jensen, Matthias Lutolf, Prisca Liberali, Jason R. Spence, Nikolche Gjorevski, Joep Beumer, Barbara Treutlein, Fabian J. Theis, J. Gray Camp

**Affiliations:** 1 Institute of Human Biology (IHB), Roche Pharma Research and Early Development, Roche Innovation Center, Basel, Switzerland; 2 Department of Computational Health, Institute of Computational Biology, Helmholtz Center Munich, Munich, Germany; 3 School of Life Sciences, Technical University of Munich, Munich, Germany; 4 Biozentrum, University of Basel, Basel, Switzerland; 5 Roche Innovation Center Basel, Roche Pharma Research and Early Development, Basel, Switzerland; 6 Department of Internal Medicine, Division of Gastroenterology and Hepatology, University of Michigan Medical School, Ann Arbor, MI USA; 7 IIIrd Medical Department, Klinikum rechts der Isar, Munich, Germany; 8 School of Medicine, Technical University of Munich, Munich, Germany; 9 School of Computation, Information and Technology, Technical University of Munich, Munich, Germany; 10 Department of Biosystems Science and Engineering, ETH Zürich, Basel, Switzerland; 11 Friedrich Miescher Institute for Biomedical Research (FMI), Basel, Switzerland; 12 Novo Nordisk Foundation Center for Stem Cell Medicine, reNEW, University of Copenhagen, Copenhagen, Denmark; 13 Laboratory of Stem Cell Bioengineering, École Polytechnique Fédérale de Lausanne, Lausanne, Switzerland; 14 Department of Cell and Developmental Biology, University of Michigan Medical School, Ann Arbor, MI USA; 15 Department of Biomedical Engineering, University of Michigan College of Engineering, Ann Arbor, MI USA

**Keywords:** Stem cells, Genome informatics, Organogenesis, Stem-cell research, Functional genomics

## Abstract

Human pluripotent stem cells and tissue-resident fetal and adult stem cells can generate epithelial tissues of endodermal origin in vitro that recapitulate aspects of developing and adult human physiology. Here, we integrate single-cell transcriptomes from 218 samples covering organoids and other models of diverse endoderm-derived tissues to establish an initial version of a human endoderm-derived organoid cell atlas. The integration includes nearly one million cells across diverse conditions, data sources and protocols. We compare cell types and states between organoid models and harmonize cell annotations through mapping to primary tissue counterparts. Focusing on the intestine and lung, we provide examples of mapping data from new protocols and show how the atlas can be used as a diverse cohort to assess perturbations and disease models. The human endoderm-derived organoid cell atlas makes diverse datasets centrally available and will be valuable to assess fidelity, characterize perturbed and diseased states, and streamline protocol development.

## Abstract (web summary)

The human endoderm-derived organoid cell atlas (HEOCA) presents an integrative analysis of single-cell transcriptomes across different conditions, sources and protocols. It compares cell types and states between models, and harmonizes cell annotations through mapping to primary tissues.

## Main

In vitro human biosystems that model complex aspects of human tissues in controlled conditions can be used as inroads into human-specific biology and disease, as well as accurate alternatives to animal models^1^. The term organoid is a current nomenclature to describe three-dimensional (3D) cell cultures derived from pluripotent, fetal or adult stem cells (PSCs, FSCs, ASCs) that recapitulate important aspects of cell composition, cytoarchitecture and functional properties of the tissue counterpart^2^. However, variations in protocols, culture conditions and stem cell sources make it challenging to assess how well organoid-derived cell states and interactions reflect those in vivo. In addition, the lack of centralized datasets and inconsistent protocol reporting complicate comparisons across studies, making it difficult to evaluate organoid fidelity, identify off-target or missing cell types, and predict genetic drivers of differentiation^3^. Overcoming these obstacles could help to better understand how human cell types and states develop, as well as support opportunities for translational research^4,5^. Advances in technology have led to the growth of single-cell transcriptome datasets, both in terms of dataset size and quantity. This has prompted collaborations to create extensive reference atlases for adult and developing human organs^4–8^. Organoids offer the opportunity to deepen our understanding of health and disease, by providing avatars of diverse developmental stages, genetic variation and disease states that will complement primary tissue atlases^5^. However, the scale of generating a comprehensive organoid atlas in individual research groups is currently impractical. Therefore, the integration of datasets generated by the wider research community becomes crucial.

The endoderm contributes to the development of the epithelial lining of a variety of different organs including thyroid, esophagus, lung, pancreas, liver, biliary system, stomach, small intestine and colon^9^. Complex endodermal 3D organoids can be differentiated from IPSCs, FSCs and ASCs in media supplemented with growth factors that promote stem cell proliferation and differentiation^10,11^, potentially enabling exploration of human ontogenetic processes of each tissue^12,13^. Here, we present an integrated single-cell transcriptomic atlas of human endoderm-derived organoids encompassing nine different tissues, combining newly generated data and data from 55 publications. We applied the atlas as a diverse cohort to assess organoid protocols, perturbations and disease models.

## Results

### Data integration to construct the organoid atlas

**Fig. 1:**
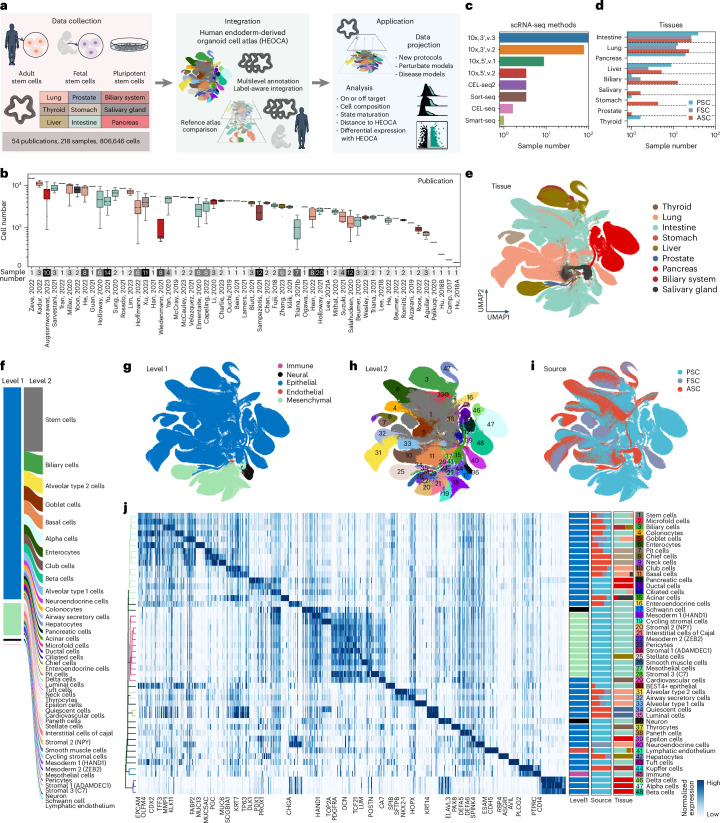
Integrated transcriptome cell atlas of human endoderm-derived organoids. **a**, Schematic overview of the atlas integration and downstream analyses. **b**, Box plot of cell number in samples from all publications, with sample number indicated below. The center represents the median; bounds indicate 25% and 75% percentiles; and whiskers show minimum and maximum values within 1.5 times the interquartile range. **c**,**d**, Bar plot showing the number of samples grouped by different single-cell sequencing methods (**c**) and by tissue and stem cell source organoid (**d**). **e**, UMAP of the organoid atlas colored by tissue. **f**, Overview of level 1 and level 2 cell annotations and cell proportion. **g**–**i**, Organoid atlas by level 1 annotations (**g**), level 2 annotations (**h**) or by stem cell source (**i**). **j**, Heatmap showing marker gene expression for each level 2 cell type in the atlas. Side stacked bar plots show proportions of cell types at level 1, stem cell source and tissue type annotations.

To create an endoderm-derived organoid cell atlas, we assembled single-cell RNA sequencing (scRNA-seq) and single-nucleus RNA sequencing data from 54 published datasets and a newly generated dataset (45,281 cells, 11 samples, small and large intestine, stomach and liver organoids) (Fig. 1a and Supplementary Table 1). Together, these datasets include samples from 218 experiments conducted on organoid models of 9 different organs (lung, liver, biliary system, stomach, pancreas, small and large intestine, prostate, salivary glands) (Fig. 1a,b). Data were obtained using multiple sequencing protocols, including plate-based methods such as Smart-seq, CEL-seq and Sort-seq, as well as commercialized droplet-based methods (for example, 10x Genomics) (Fig. 1c). Based on availability, we incorporated organoid datasets that model healthy states primarily of human endoderm-derived tissues, with source material from PSCs (embryonic stem cells and induced PSCs), FSCs or ASCs (Fig. 1d). Notably, we obtained data of each stem cell source from intestine, lung, liver and biliary system organoid models (Fig. 1b,d). In total, we collected 806,646 cells to be utilized for downstream integration and analysis (Fig. 1a–d).

**Extended Data Fig. 1:**
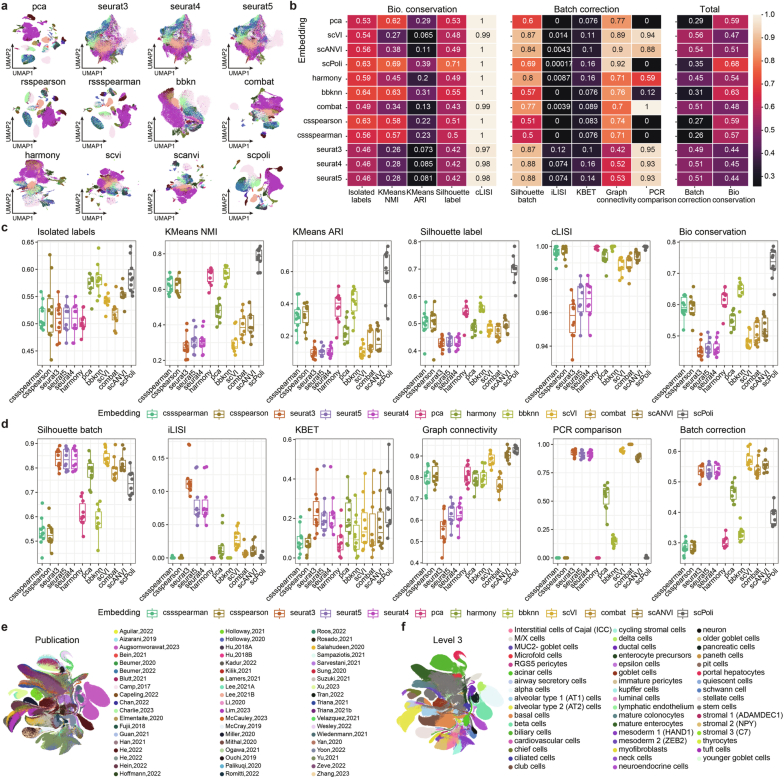
Analysis of scRNA-seq integration methods. (**a**) Example UMAP of tested integration methods and without any data integration (PCA). Dots in all UMAP embeddings are colored by the level 2 cell type annotation. (**b**) Example scIB benchmarking metrics for all tested integration methods. (**c**) The boxplot displays the benchmarking results for ten scIB biology conservation tests. (**d**) The boxplot shows the benchmarking results for ten scIB batch correction tests. (**e-f**) UMAP of the organoid atlas colored by publications (**e**) and level 3 cell annotations (**f**). For boxplots in c,d, p values from two-tailed Mann–Whitney U test; 10 random repeats were performed; the center of the box represents the median; the bounds of the box indicate the 25% and 75% percentiles, while the whiskers show the minimum and maximum values within 1.5 times the interquartile range.

**Extended Data Fig. 2:**
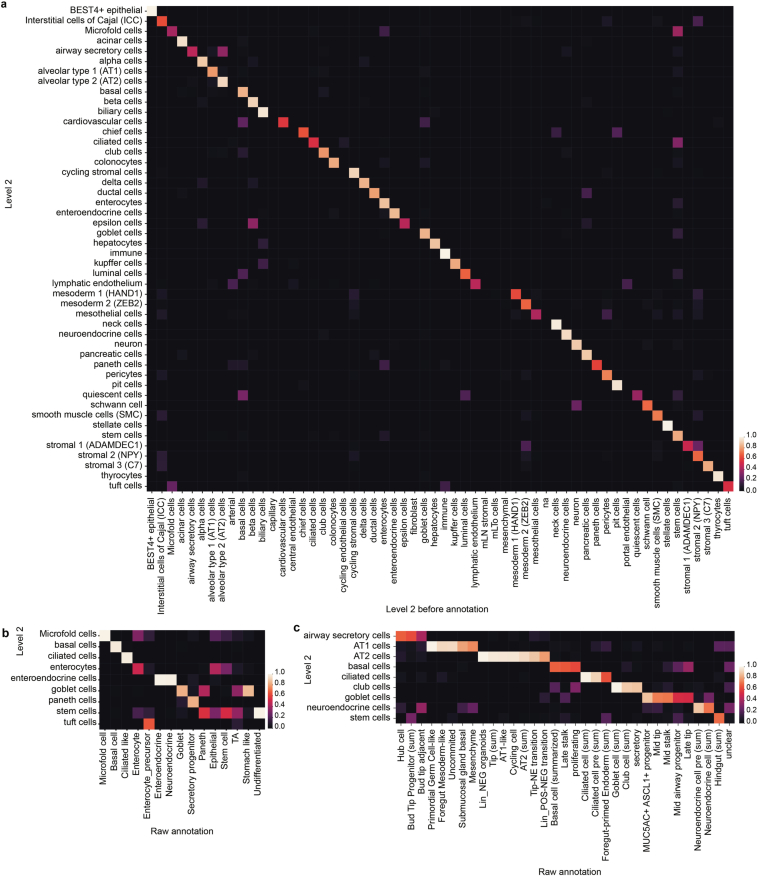
Cell type annotation comparison between before and after integration. (**a**) Heatmap depicting sample based annotation and post-integration annotation overlap. (**b**) Heatmap depicting HIOCA annotations and author annotation overlap normalized by column. (**c**) Heatmap depicting HLOCA annotations and author annotation overlap normalized by column.

**Extended Data Fig. 3:**
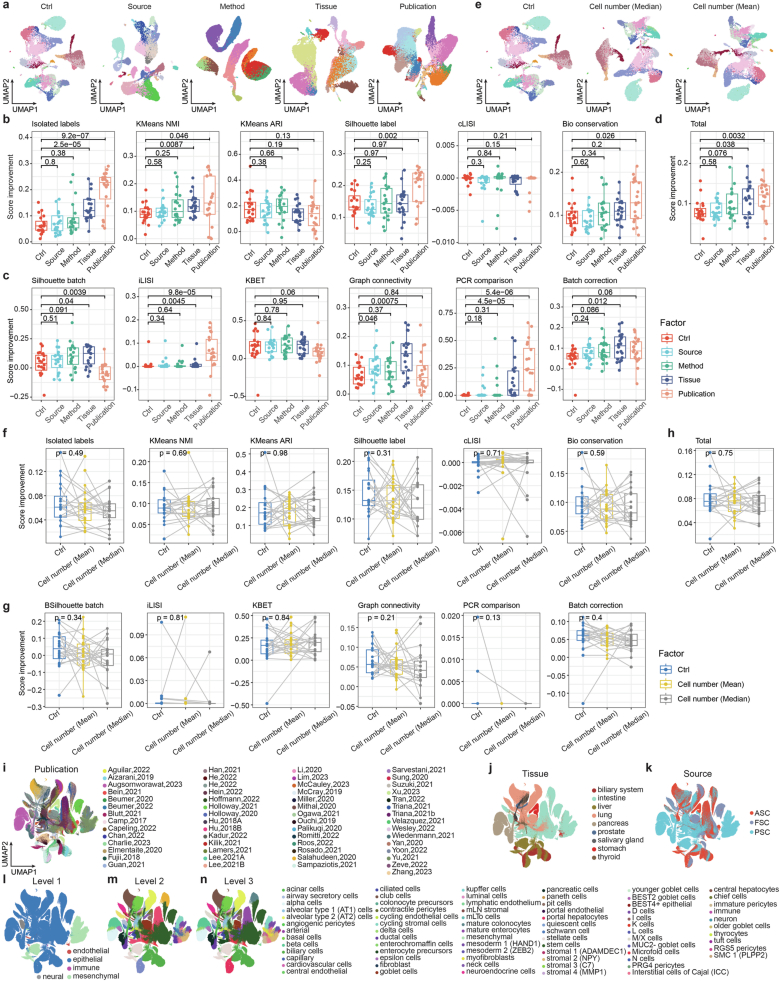
Analysis of how scRNA-seq integration methods are affected by sample variances. (**a**) UMAP example showing samples with identical factor variances integrated using the scPoli method. (**b**) Boxplot depicting the results of 20 scIB biology conservation benchmarks with consistent factor variances. (**c**) Boxplot showing the results of 20 scIB batch correction benchmarks with the same factor variances. (**d**) Boxplot summarizing the total benchmarking results for scIB biology conservation and batch correction, with consistent factor variances. (**e**) UMAP example illustrating samples with a subset of cell number variance integrated using the scPoli method. (**f**) Boxplot presenting the results of 20 scIB biology conservation benchmarks for samples with a subset of cell numbers. (**g**) Boxplot showing the results of 20 scIB batch correction benchmarks for samples with a subset of cell numbers. (**h**) Boxplot summarizing the total benchmarking results for scIB biology conservation and batch correction with a subset of cell numbers. (**i-n**) UMAPs of integration with a median subset of cell numbers, colored by (**i**) publication, (**j**) tissue, (**k**) stem cell source, (**l**) level 1 annotation, (**m**) level 2 cell annotations, and (**n**) level 3 cell annotations. For boxplots in b,c,d,f,g,h, 20 random repeats were performed; p values from two-tailed Mann–Whitney U test; the center of the box represents the median; the bounds of the box indicate the 25% and 75% percentiles, while the whiskers show the minimum and maximum values within 1.5 times the interquartile range.

**Extended Data Fig. 4:**
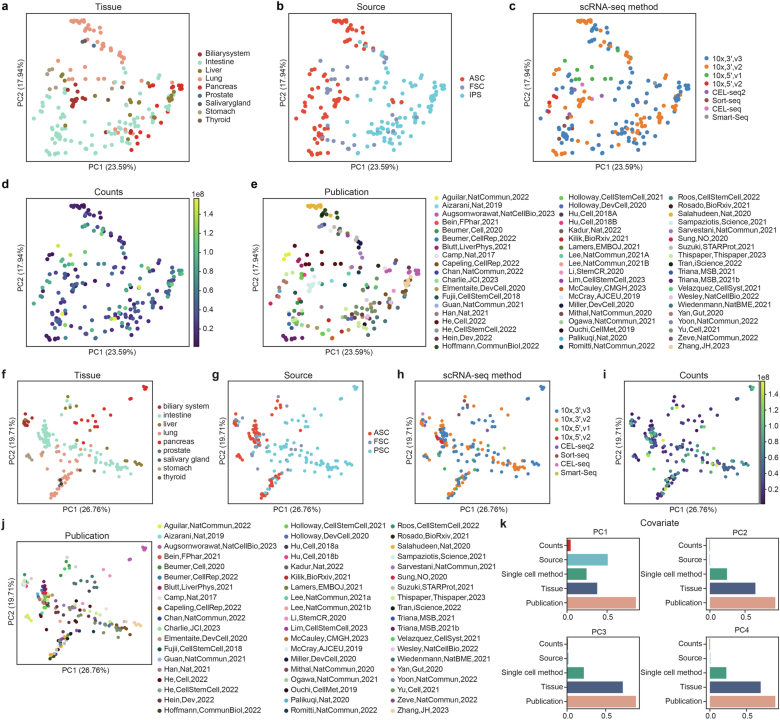
Pseudo-bulk analysis was performed on all collected organoid scRNA-seq data, using both raw and scPoli embedding datasets. (**a-e**) PCA plot showing pseudo-bulk transcriptomes from each organoid single-cell RNA-seq sample, colored by: (**a**) tissue type, (**b**) stem cell source, (**c**) scRNA-seq methods, (**d**) total counts in the sample, and (**e**) publication. (**f-j**) PCA plot showing pseudo-bulk scPoli embedding all organoid samples, colored by: (**f**) tissue type, (**g**) stem cell source, (**h**) scRNA-seq methods, (**i**) total counts in the sample, and (**j**) publication. (**k**) The bar plots display the adjusted correlation coefficients between sample variance factors (such as sample counts, stem cell source, scRNA-seq method, tissue type, and publication) and scPoli embedding principal components (from left to right: PC1 to PC4).

We clustered cells at high resolution in each dataset and assigned cell annotations based on known marker gene expression and differential expression between clusters (Supplementary Table 2). To assist with label-aware integration, we established a three-level hierarchical cell-type annotation: class (level 1), type (level 2) and subtype (level 3) (Extended Data Fig. 1). To address batch effects and achieve a robust atlas integration, we assessed 12 different data-integration methods using single-cell integration benchmarking^14–21^ (Extended Data Fig. 1a–d), and selected scPoli^20,22^ to generate an integrated embedding of all organoid cells, enabling a cohesive representation of the diverse data (Fig. 1e and Extended Data Fig. 1e). The integrated atlas was reannotated based on the most frequent cell type in each cluster, resulting in 5 cell classes at level 1, 48 cell types at level 2 and 51 cell subtypes at level 3 (Fig. 1f–h and Extended Data Fig. 1f). Comparing annotations before and after integration with annotations in the original manuscripts showed a high consistency across most cell-type labels (Extended Data Fig. 2a–c). Inconsistencies were related to states on continuous differentiation trajectories and nomenclature granularity between publications (Supplementary Table 3). Integration performance was unaffected by stem cell source, single-cell method or tissue type, but dataset origin substantially influenced integration outcomes (Extended Data Fig. 3 and Supplementary Table 4). Pseudo-bulk analysis using both raw and scPoli embedding on all organoid single-cell datasets revealed stem cell source and tissue type as primary drivers of variance (Extended Data Fig. 4).

Overall, epithelial cells from different organs clustered together in the integrated atlas and clusters were composed of cells from different stem cell sources (Fig. 1e,i). However, we also identified cell types with contributions from multiple organoid models. For example, goblet cells were found in both intestine (68.08%) and lung (31.84%), with a minor presence in other organs (0.08%). Basal cells were observed in the lung (71.29%), salivary gland (16.28%), intestine (10.41%) and thyroid (1.32%) models (Fig. 1j). These results suggest the existence of cell types that exhibit partial or shared characteristics across different organ models, and also may indicate off-target cells in organoids. We identified consistent markers for each integrated cell type across datasets and protocols, such as *OLFM4* for stem cells and *TP63* for basal cells (Fig. 1j and Supplementary Table 5). We note instances in which cells derived from organoid models of a certain organ clustered with cells annotated as being from a different organ. Given the difficulty in precisely controlling organoid development, especially PSC-derived organoids, off-target cells in organoids are a known issue^23^. In addition, organoids derived from primary FSCs or ASCs could be contaminated because of handling or the adjacency of tissues during tissue acquisition, or cell states could be different from the tissue of origin because of stem cell plasticity. Therefore, it is important to develop strategies to compare organoid cells with reference counterparts.

### Reference atlas comparison to assess organoid fidelity

**Fig. 2:**
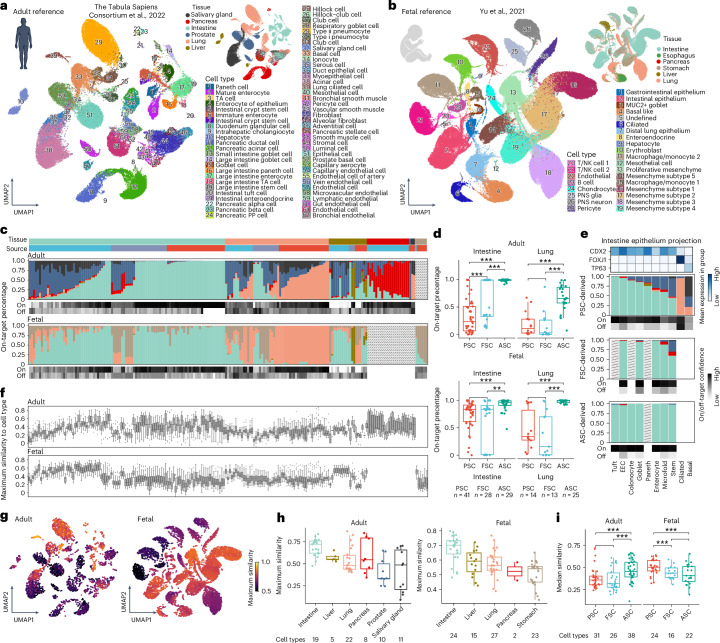
Mapping organoid cell types to a primary tissue reference atlas to assess organoid fidelity. **a**,**b**, UMAP representations of an integrated object comprising primary adult (**a**) and fetal (**b**) cell types and tissues are shown in the top right, as presented in the original publication. **c**, Bar plots showing the tissue proportion of the most similar adult (top) and fetal (bottom) tissue, sorted by organoid tissue and stem cell source. The upper annotation bar indicates the organoid tissue and stem cell source, with tissue colors matching Fig. 1e and stem cell source colors matching Fig. 1i. Matching tissue and organoid indicate on-target, whereas mismatched tissue and organoid indicate off-target. Scaled color bars at the bottom of the bar plots represent the mean confidence of on-target and off-target cells. Missing reference samples are depicted as a gray bar with a black line. **d**, Box plots reveal the percentage of on-target cells in intestine and lung organoid samples for adult (top) and fetal (bottom) cells. **e**, Similar to **c**, a subset of all intestine organoid epithelium cells projected to adult tissue is split by the source of stem cells. On- and off-target confidence is shown at the bottom of each bar plot, with three marker gene expressions in corresponding cell types shown at the top. **f**, Box plots illustrate the highest similarity of all cell types in the corresponding primary adult and fetal tissues for each organoid sample, sorted as shown in **c**. **g**, UMAPs for primary adult (left) and fetal (right) tissues demonstrate the maximum similarity of all organoids in the comprehensive cross-tissue organoid atlas. **h**, Box plots show the maximum similarity of each adult (left) and fetal (right) cell type in different tissues. **i**, Box plots present the median similarity to primary adult (left) and fetal (right) cell types among different sources of stem cell organoids. For plots in **d**, **f**, **h** and **i**, *P* values are from two-tailed Mann–Whitney *U*-tests. In box plots, the center represents the median; bounds show the 25% and 75% percentiles; and whiskers indicate values within 1.5× the interquartile range. EEC, enteroendocrine cell; NK, natural killer; PP, pancreatic polypeptide; TA, transit-amplifying.

**Extended Data Fig. 5:**
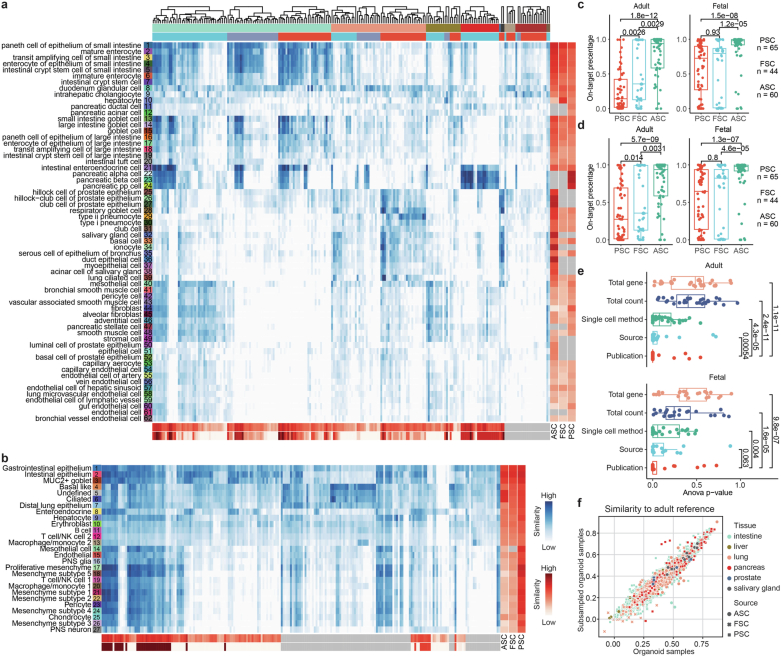
Organoid cell type comparison to cell types in fetal and adult primary tissue atlases. (**a-b**) Heatmaps display the similarity between each organoid sample and all adult primary tissue (**a**) and fetal primary tissue (**b**) cell types. The upper sidebar annotations indicate the organoid tissue and the source of stem cells. The right-hand sidebar annotations display the maximum similarity of different stem cell sources within their corresponding tissues. The lower sidebar annotations depict the average similarity of all cell types in corresponding tissues within each organoid sample. (**c**) Box plots reveal the percentage of on-target cells in all organoid samples using adult (left) and fetal (right) tissue as a reference, ordered as PSC, FSC, and ASC from left to right. (**d**) Similar to (**c**), but showing the subset of organoid epithelial cells. (**e**) The boxplot shows the covariance of single-cell factors (publications, source of stem cells, scRNA-seq methods, total sample counts, and total gene counts) with the adult and fetal primary tissue comparisons. Biological sample size: adult = 37; fetal = 24. (**f**) The scatterplot shows the correlation between the similarity of median subsampled organoid samples to adult primary tissue and the overall similarity of organoid samples to adult primary tissue. For box plots in c,d,e, the center of the box represents the median; the bounds of the box indicate the 25% and 75% percentiles, while the whiskers show the minimum and maximum values within 1.5 times the interquartile range.

To evaluate the fidelity of cell states observed in the human endoderm-derived organoid cell atlas (HEOCA), we obtained published scRNA-seq data on human endoderm-derived organs from adult (small and large intestine, lung, liver, pancreas, prostate, salivary gland)^6^ (Fig. 2a) and fetal (small and large intestine, lung, liver, pancreas, stomach, esophagus)^23^ (Fig. 2b) specimens. To assess on- and off-target cells in organoids, we projected organoid cells to the fetal and adult primary tissue atlases, and inferred the target tissue via label transfer (Fig. 2c). PSC-derived organoids have a lower on-target percentage in both fetal and adult primary tissues compared with FSC- and ASC-derived organoids (Fig. 2c and Extended Data Fig. 5a–d). Focusing on intestine and lung organoids, FSC- and ASC-derived intestine organoids demonstrated high on-target percentages, with an average of 91.12% in FSC-derived organoids and 98.14% in ASC-derived organoids (Fig. 2d). By contrast, PSC-derived organoids displayed a median on-target percentage of between 23.28% and 83.63% depending on fetal or adult reference atlas comparison; however, this is likely a low estimate because datasets from early organoid time points are difficult to assess using this reference comparison (Fig. 2c–e).

We identified major cell types from each adult and fetal tissue (Fig. 2a,b), and compared organoid cell types and states with primary counterparts using neighborhood graph correlation^24^. We quantified the proportion of cell types in each organoid sample and compared the similarity of each cell type with counterparts in adult and fetal tissues (Fig. 2f–h and Extended Data Fig. 5a,b). ASC-derived organoids had the highest similarity to adult counterparts, whereas PSC-derived organoids were most similar to fetal counterparts, with FSC-derived organoid cell states showing an intermediate distribution (Fig. 2i). Multiple regression analyses revealed that similarity to reference atlases was influenced by publication and stem cell source but not by scRNA-seq methods, total sample counts or total sample genes (Extended Data Fig. 5e,f).

### Intestinal organoid atlas covers development and adult biology

**Fig. 3:**
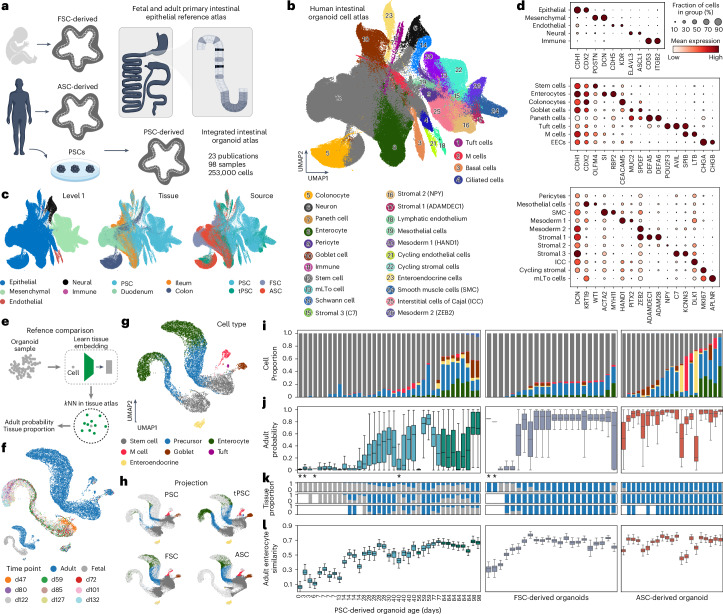
Human intestinal organoids from different stem cell origins generate developing and adult cell states. **a**, Analytical design of the intestine organoid subatlas and comparison with the primary reference tissue. **b**,**c**, UMAP of healthy intestinal organoid atlas colored by level 1 cell-type annotation, source of intestine tissues and source of stem cells (**b**) and level 2 cell-type annotation (**c**). **d**, Dot plots showing intestinal marker gene expression across organoid cell types. From top to bottom, the dot plots display level 1 cell markers, epithelial cell markers and mesenchymal cell markers. **e**, Analytical design of the intestine organoid subatlas and comparison with the primary reference tissue. **f**,**g**, UMAP of the integrated intestine fetal and adult primary tissue single-cell object colored by adult sample or fetal sample age (**f**) and cell type (**g**). **h**, Projection of intestine organoid cells onto fetal and adult primary epithelial single-cell objects categorized by PSC-, transplant PSC- (tPSC), FSC- and ASC-derived organoid samples. **i**, Bar plot illustrating the predicted cell proportions of each organoid sample mapped to the primary tissue objects. The samples are divided by PSC-, FSC- and ASC-derived organoid samples, with PSC-derived organoids further ordered by organoid age, and FSC- and ASC-derived organoids ordered by the percentage of stem cells. **j**, Box plot showing the predicted probability of cell mapping to adult samples. The cell numbers range from 1 to 10,866, with samples containing fewer than 100 cells marked by an asterisk. **k**, Bar plots illustrating the predicted tissue (fetal in gray and adult in blue) proportions. From top to bottom are stem cells, precursor enterocytes and enterocytes. **l**, Box plot showing the adult enterocytes similarity of each organoid sample. The order of organoid samples in **j**, **k** and **l** is consistent with that in **i**. Biological sample size is 163. For the box plots in **j** and **l**, the center represents the median; bounds show the 25% and 75% percentiles; and whiskers indicate values within 1.5× the interquartile range. d, day; mLTo, mesenchymal lymphoid tissue organizer.

**Extended Data Fig. 6:**
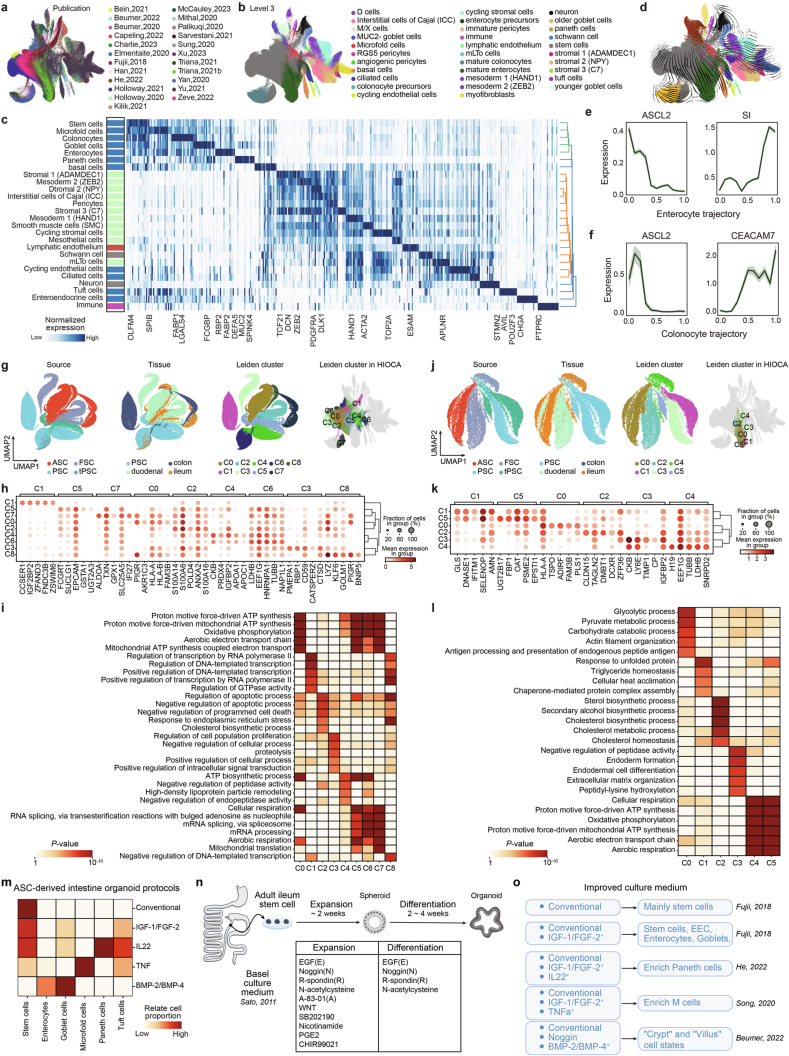
Integrated intestine organoid atlas analysis and comparison to primary intestine tissue. (**a-b**) UMAP of the intestine organoid atlas colored by publications (**a**) and level 3 cell annotations (**b**). (**c**) Heatmap showing marker gene expression for each level 2 cell type in the intestine organoid atlas. Side stacked barplots show proportions of cell types at level 1 annotation. (**d**) UMAP of the integrated intestine organoid atlas with cells colored according to level 2 annotations. The stream arrows visualize the inferred velocity flow of cell states, providing insights into cellular dynamics. (**e**) Expression profiles along the pseudotime trajectory from stem cells to enterocytes of ASCL2 (stem cell) and SI (enterocytes). The error bar indicated the 95% confidence interval for the regression estimate. (**f**) Expression profiles along the pseudotime trajectory from stem cells to colonocytes of ASCL2 (stem cell) and CEACAM7 (colonocytes). The error bar indicated the 95% confidence interval for the regression estimate. (**g**) UMAP of intestinal organoid atlas stem cells, colored by stem cell source, tissue type, and Leiden cluster (from left to right). (**h**) Dotplot showing the top 5 marker genes for each Leiden cluster of stem cells in (**g**). (**i**) Heatmap illustrating GO enrichment analysis of differentially expressed genes across the Leiden clusters of stem cells shown in (**g**). The p-value was computed using Fisher's exact test. (**j**) UMAP of intestinal organoid atlas enterocytes, colored by stem cell source, tissue type, and Leiden cluster (from left to right). (**k**) Dotplot showing the top 5 marker genes for each Leiden cluster of enterocytes in (**j**). (**l**) Heatmap illustrating GO enrichment analysis of differentially expressed genes across the Leiden clusters of enterocytes shown in (**j**). The p-value was computed using Fisher's exact test. (**m**) The heatmap illustrates the relationship between summarized factors or protocols and the proliferation of epithelial cell types. (**n**) A schematic diagram illustrating the ASC-derived small intestine organoid protocol and the conventional basal culture medium. (**o**) A summary of the improved culture medium based on the conventional basal culture medium.

To explore organoid cell states of different stem cell origin, we focused on intestinal organoid models in which there is substantial coverage from PSC-, FSC- and ASC-derived organoid cells. This subset consisted of 98 samples from 23 different publications representing 353,140 single-cell transcriptomes (Fig. 3a, Extended Data Fig. 6a and Supplementary Table 1). We reintegrated all cells and defined 5 cell types at level 1, 26 cell types at level 2 and 32 cell types at level 3 in the atlas (Fig. 3b,c and Extended Data Fig. 6b,c). This integrated intestinal organoid atlas (HIOCA) covers epithelial states from the duodenum, ileum, colon and PSC-derived organoids, and contains a large fraction of mesenchymal cells, and minor populations of neural, endothelial and immune cell types (Fig. 3b,c and Extended Data Fig. 6d–f). We subsetted and reintegrated stem cells and enterocytes, and found that cells from different sources or tissues clustered together and exhibited distinct gene expression profiles (Extended Data Fig. 6h,i). We used the large collection of protocols to examine factors that influence cell-type proportion (Extended Data Fig. 6m–o). For instance, tumor necrosis factor (TNF) and interleukin-22 (IL-22) are linked to more abundant microfold (M) and Paneth cells in ASC-derived organoids, respectively, and xenografted PSC-derived tissues harbor both Paneth and tuft cells, which are absent in early stage PSC-derived organoids. Protocol evaluation suggests tailored approaches to enrich specific cell types or enhanced maturation (Extended Data Fig. 6o).

**Extended Data Fig. 7:**
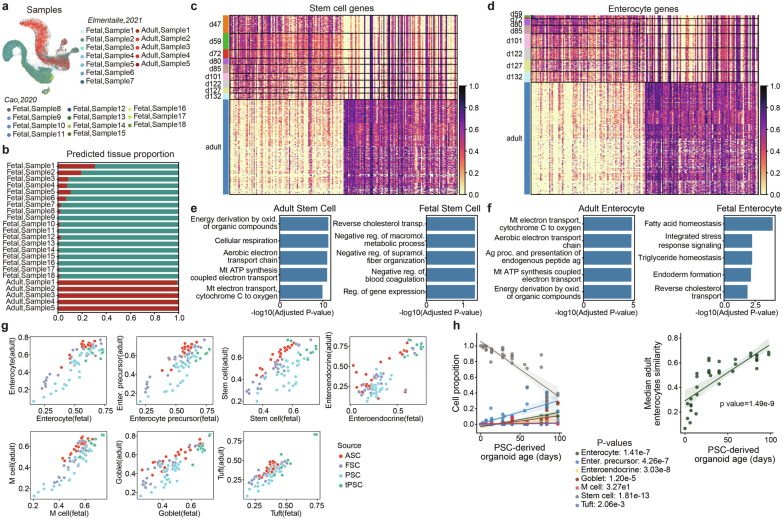
Integrated primary intestine tissue features. (**a**) The UMAP visualization displays 18 fetal tissue samples and five adult tissue samples, originating from two publications, projected onto fetal and adult primary tissue single-cell objects, with cells colored according to different samples. (**b**) Stacked bar plots provide a visual representation of the predicted proportions of fetal and adult cells in all tissue samples. (**c**) The heatmap shows the top differentially expressed genes in fetal and adult stem cells. (**d**) Similarly, the heatmap shows differentially expressed genes in fetal and adult enterocytes. (**e**) The top five enriched GO terms for differentially expressed genes in adult and fetal stem cells. The p-value was computed using Fisher's exact test. (**f**) Similarly, the top five enriched GO terms for differentially expressed genes in adult and fetal enterocytes. The p-value was computed using Fisher's exact test. (**g**) Scatter plots illustrate the maximum fetal or adult cell type similarity across all intestinal organoid samples. (**h**) The relationship between the age of PSC-derived organoids and cell proportion and adult similarity. The error bar indicated the 95% confidence interval for the regression estimate.

To assess intestinal organoid fidelity and maturation, we integrated time series scRNA-seq data from duodenal development (59 to 132 days post fertilization) with adult intestinal epithelium^23,25,26^ (Fig. 3e). These data revealed distinct fetal and adult stem cell-to-enterocyte differentiation trajectories, while other epithelial cell types, such as goblet, tuft, M and enteroendocrine cells, showed similar states across both stages (Fig. 3f,g and Extended Data Fig. 7a–f). Comparing organoids with the primary reference revealed that PSC-derived organoids resembled fetal tissue, whereas FSC- and ASC-derived organoids aligned with adult tissues (Fig. 3h), consistent with reports that FSC-derived organoids lose fetal traits during extended culture^27^. Metrics such as cell-type proportion, projection probability and similarity to fetal and adult cell types highlighted substantial variation across samples (Fig. 3i–l and Extended Data Fig. 7g,h). For example, PSC-derived organoids increase in complexity and reference similarity over time in culture, and after xenografting into a mouse host for maturation, the organoids obtain higher cellular diversity and similarity to primary tissue differentiated enterocytes. Altogether, these results reveal the diversity of cell composition and cell maturation in intestinal organoids from different sources, time points and protocols.

### Lung organoid atlas covers development and adult biology

**Fig. 4:**
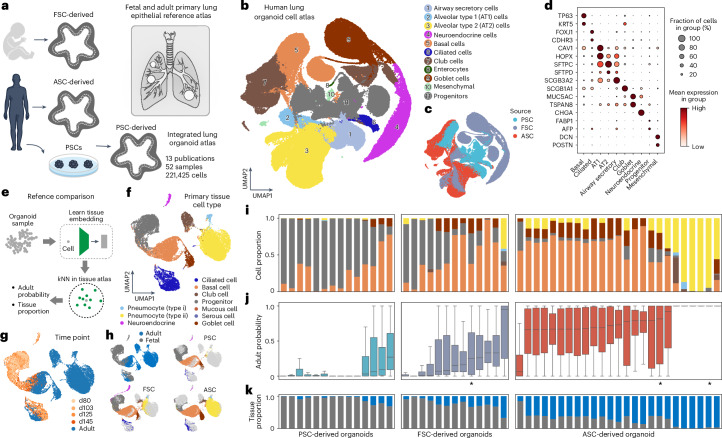
Human lung organoids from different stem cell origins generate developing and adult cell states. **a**, Schematic of the analyses performed on the lung organoid subatlas (HLOCA) and comparison with the primary reference tissue. **b**,**c**, UMAP of the integrated object of all lung organoid samples colored by cell type (**b**) and stem cell source (**c**). **d**, Dot plot showing lung marker gene expression across organoid cell types. **e**, Analytical design of the lung organoid subatlas and comparison with the primary reference tissue. **f**,**g**, UMAP of the integrated lung fetal and adult primary tissue single-cell object colored by adult sample or fetal cell type (**f**) and age of sample (**g**). **h**, Projection of lung organoid cells onto fetal and adult primary epithelial single-cell objects categorized by PSC-, FSC- and ASC-derived organoid samples. **i**, Bar plot illustrating the predicted cell proportions of each organoid sample mapped to the primary tissue objects. The samples are divided by PSC-, FSC- and ASC-derived organoid samples from left to right. **j**, Box plot showing the predicted probability of cell mapping to adult samples. The cell numbers range from 395 to 13,017, with samples containing fewer than 500 cells marked by an asterisk. The center represents the median; bounds show the 25% and 75% percentiles; and whiskers indicate values within 1.5× the interquartile range. **k**, Bar plots illustrating the predicted tissue (fetal in gray and adult in blue) proportions. The order of organoid samples in **j** and **k** is consistent with that in **i**.

We performed a detailed analysis of lung organoid cells, consisting of 221,425 cells obtained from 52 samples and 13 publications, comprising PSC-, FSC- and ASC-derived sources (Fig. 4a). We integrated, clustered and annotated these data to generate a human lung organoid cell atlas (HLOCA) (Fig. 4b,c). The Uniform Manifold Approximation and Projection (UMAP) representation showed integration of data from different publications and samples with undifferentiated stem cells positioned centrally surrounded by more differentiated cell types (Fig. 4b). Organoids from PSCs displayed a higher proportion of lowly differentiated early endoderm development marker genes such as *FABP1* and *AFP*-defined progenitor cells, which were largely absent in the ASC-derived organoids (Fig. 4d). In turn, organoids obtained via ASC-protocols frequently contained a relevant proportion of club cells, whereas a high incidence of goblet and neuroendocrine cells was primarily observed in samples produced using FSC protocols (Fig. 4b–d). Overall, the differences in cell-type composition suggest effects from stem cell source as well as details of the protocol, including media and growth factors. We provide a structured account of the publicly available metadata on lung organoid datasets in the atlas including information on all available protocol components, concentrations and intervals, which can be linked to the samples in the shared HLOCA object.

To gain insights into how the lung organoid datasets correspond with primary tissue, we integrated a unified reference of primary adult and fetal lung tissues^6,23^ (Fig. 4e–k). The query to reference mapping of the lung organoid data showed that PSC-derived organoid cells preferentially integrated with fetal counterparts, ASC-derived organoid cells integrated with adult counterparts and FSC-derived organoid cells projected to both fetal and adult references (Fig. 4h). This finding is consistent with previous observations from intestine reference mapping analysis in which PSC-derived organoids model fetal biology, ASC-derived organoids model adult biology and FSC-derived organoids have intermediate or unclear mappings. Metrics such as cell-type proportion, projection probability and similarity to fetal and adult cell types also highlighted substantial variation across samples (Fig. 4i–k). Interestingly, the results show that most PSC-derived and some FSC-derived organoids contain a large proportion of cells resembling early fetal epithelial cells. This observation is consistent with our previous finding of undifferentiated cells in PSC- and FSC-derived organoids. In summary, these data offer an integrated atlas for lung organoids (HLOCA) to complement the HEOCA for the study of lung 3D cultures at a single tissue level, providing insight into differences in cell-type composition, maturation state and resemblances to primary tissue from multiple stem cell sources.

### Protocol assessment and projection of new data

**Fig. 5:**
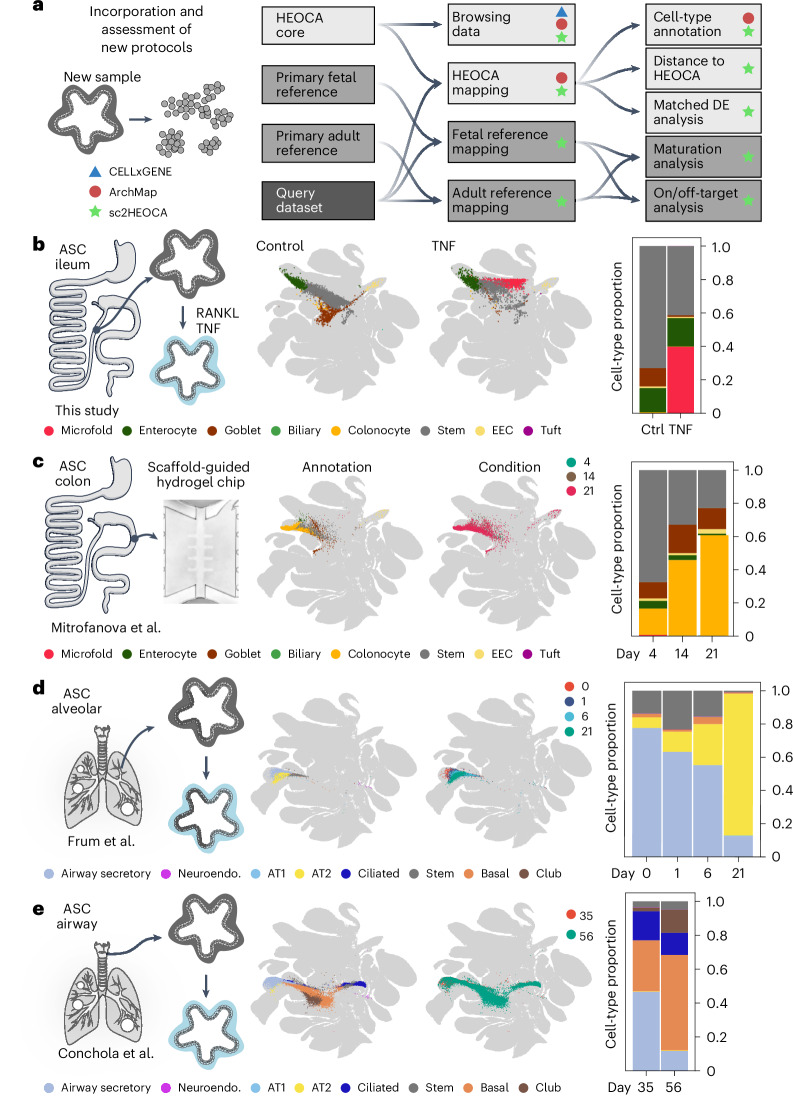
The integrated atlas enables protocol assessment and can be extended via dataset projection. **a**, Schematic representation showing the analytical pipelines and varied interfaces to facilitate analyzing scRNA-seq data of organoid samples for the atlas. **b**, Experimental design of the ileum organoid sample with TNF treatment to generate M cells. The UMAP of sample scRNA-seq data mapped to the organoid atlas is colored by predicted level 2 cell types. The bar plot shows the cell proportions of predicted level 2 cell types across control and TNF treatment scRNA-seq data. **c**, Experimental design of the colon organoid sample using a scaffold-guided hydrogel chip model. The UMAP of sample scRNA-seq data mapped to the organoid atlas is colored by predicted level 2 cell types and time points, with a bar plot depicting the cell proportions of predicted level 2 cell types across the time course scRNA-seq data. **d**, Experimental design of the lung alveolar organoid samples. The UMAP of sample scRNA-seq data mapped to the organoid atlas is colored by predicted level 2 cell types and time points, with a bar plot depicting the cell proportions of predicted level 2 cell types across the time course scRNA-seq data. **e**, Experimental design of the lung airway organoid samples. The UMAP of sample scRNA-seq data mapped to the organoid atlas is colored by predicted level 2 cell types and time points, with a bar plot depicting the cell proportions of predicted level 2 cell types across the time course scRNA-seq data. Ctrl, control; DE, differential expression; Neuroendo., neuroendocrine.

We developed a toolkit to incorporate organoid datasets and compare data with cell states in the integrated HEOCA (Fig. 5a). This toolkit (sc2heoca) offers functions to compare samples with tissue references and assess ‘on or off’ target status and cell state maturation. In addition, it enables sample projection onto the integrated HEOCA through nearest neighbor analysis and cell annotation through label transfer. The mean expression of the nearest neighbors serves as paired reference cells for differential expression analysis and mean distance to nearest neighbors provides an estimate for the level of difference between sample and reference states. We applied this toolkit to assess organoid protocols, perturbations and disease models (Fig. 5a).

**Extended Data Fig. 8:**
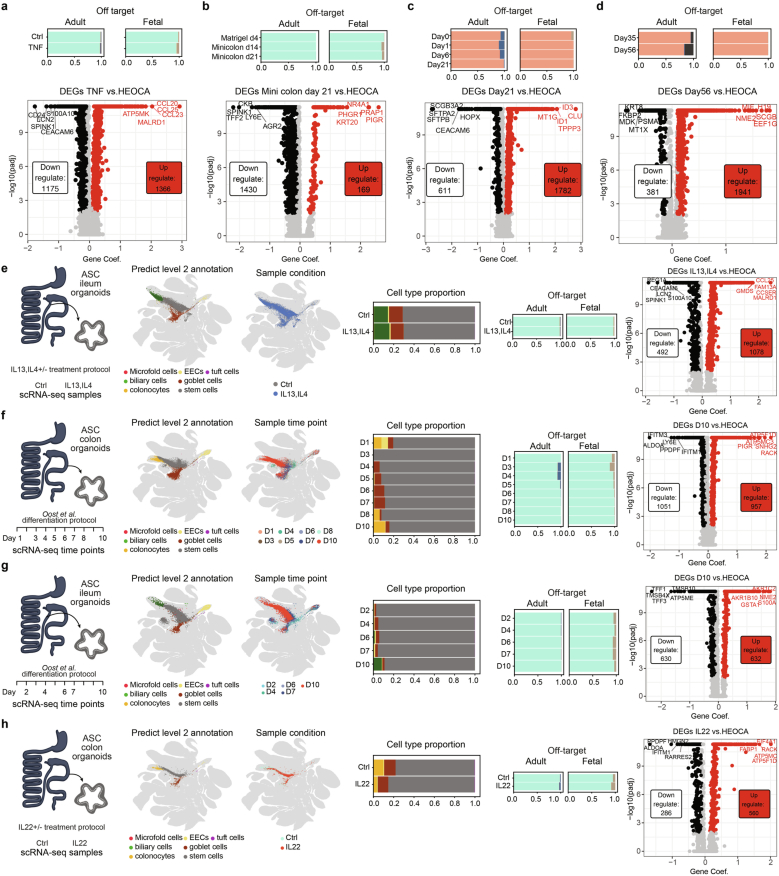
Integration and extended analysis of organoid protocol datasets. (**a**) Ileum organoid samples treated with TNF to promote Microfold cell proliferation. On/Off-target bar plots display the proportions of predicted cell types targeting primary tissues, with colors matching those in Fig. 2a and b. The scatter plot illustrates differentially expressed genes between the treatment samples and HEOCA, highlighting genes upregulated in the treatment samples in red and those upregulated in HEOCA in black. (**b**) Similar analysis to (**a**) for the colon organoid sample using a scaffold-guided hydrogel chip model. (**c**) Similar analysis to (**a**) for the lung alveolar organoid samples. (**d**) Similar analysis to (**a**) for the lung airway organoid samples. (**e**) Experimental design for IL13 and IL4 treatment ileum organoid samples. The UMAP visualization of scRNA-seq data is mapped to the organoid atlas and colored by predicted level 2 cell types and time points, with a bar plot showing the proportions of these cell types over the time course. On/Off-target bar plots display the proportions of predicted cell types targeting primary tissues, with colors matching those in Fig. 2a and b. The scatter plot illustrates differentially expressed genes between the treatment samples and HEOCA, highlighting genes upregulated in the treatment samples in red and those upregulated in HEOCA in black. (**f**) Similar analysis to (**e**) for the time course colon organoid sample. (**g**) Similar analysis to (**e**) for the time course ileum organoid sample. (**h**) Similar analysis to (**e**) for the IL22 treatment colon organoid sample. The p-values in a-h were computed using the F test.

We provide several examples of how the HEOCA can be used to evaluate single-cell transcriptome datasets from recent organoid protocols (Fig. 5b–e). First, we validate a finding^28^ that modulation of the TNF pathway promotes M cell abundance in intestinal organoids (Extended Data Fig. 6m). We generated ASC-derived ileal organoids in control media or media supplemented with TNF and receptor activator of nuclear factor-κB ligand (RANKL), and performed scRNA-seq after 6 days of treatment. Reference comparison revealed that the majority of cells from both the control and TNF treatment samples accurately matched the intended intestinal tissue cell types (control, 98.26%; TNF, 95.16%) (Extended Data Fig. 8a). Projection onto the HEOCA confirmed a notable increase in M cells in the TNF treatment versus control samples, rising from 0% to 34.92%, with corresponding differential expression profiles (Fig. 5b and Extended Data Fig. 8a).

Second, we assessed colonic epithelial tissue generated by seeding human colon ASC-derived organoids on a scaffolded hydrogel in a fluidic chip^29^ (Fig. 5c). Projection analysis demonstrated that this protocol led to colonocyte differentiation and maturation, as indicated by a substantially higher proportion of colonocytes compared with the control samples (day 4, 20.45%; day 14, 54.03%; day 21, 66.97%) (Fig. 5c and Extended Data Fig. 8b). This on-chip protocol offers advantages over conventional organoid protocols by providing access to the apical and basal sides of the epithelium and allowing the culture to be maintained for many weeks while sustaining both stem and differentiated cell types.

Third, we analyzed two lung datasets consisting of time courses of lung progenitor organoids differentiated into alveolar or airway organoids (Fig. 5d,e). In the alveolar dataset, cells showed increased mapping to alveolar epithelial identities (AT1 and AT2) over the course of differentiation (Fig. 5d). This increase was accompanied by a decrease in cells mapping to undifferentiated identities in the reference atlas (Extended Data Fig. 8c). Similarly, lung progenitor organoids differentiated toward the airway were accurately mapped to airway-specific cell identities, including SCGB3A2^+^ airway progenitors, basal cells and secretory cells, consistent with previous descriptions of these organoids^30,31^ (Fig. 5e and Extended Data Fig. 8d). Notably, these cells were minimally mapped to alveolar epithelial identities, further validating the accuracy of the reference atlas in distinguishing different lung cell types (Fig. 5d,e).

Finally, we incorporated four additional ASC-derived intestinal organoid datasets (two published and two unpublished) including condition versus control ileum organoids treated with IL-4 and IL-13 and colon organoids treated with IL-22, and time course data of ileum and colon organoids in a medium to promote differentiation^32,33^. For each dataset we projected to the HEOCA, annotated cell types, assessed cell-type proportion, mapped to adult and fetal references, and performed differential expression analysis (Extended Data Fig. 8e–h). Taken altogether, these data provide a framework for protocol assessment and dataset incorporation into an integrated organoid cell atlas.

### Perturbation and disease models expand organoid cell states

**Fig. 6:**
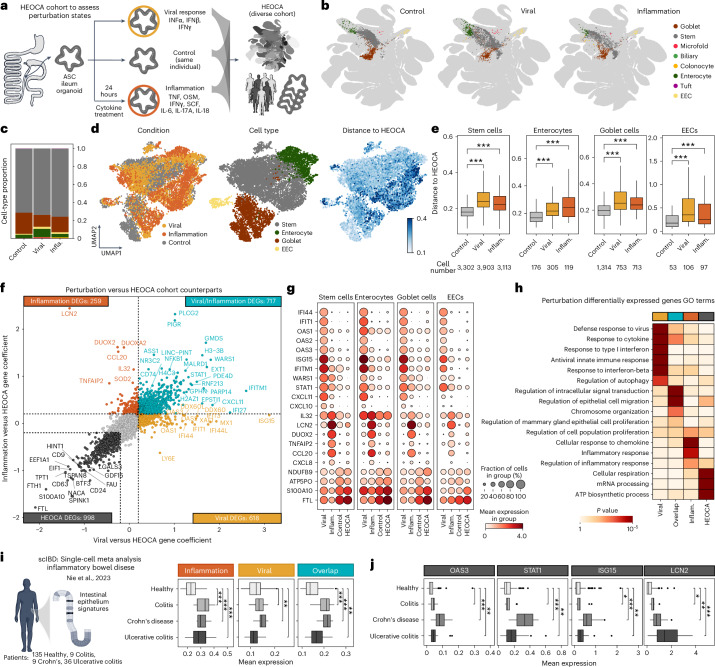
Organoid perturbation and comparison with the HEOCA extends the cell state repertoire. **a**, Summary of the cytokines used to treat ileum organoids for viral response and inflammation. The HEOCA provides a diverse cohort of cell types and states that can be used as a control for comparing perturbation conditions to reveal DEGs and under-represented cell states. **b**, UMAP of sample scRNA-seq data mapped to the HEOCA, colored by predicted level 2 cell types, from left to right: control, viral response and inflammation. **c**, Bar plot depicting the cell proportions of predicted level 2 cell types across control and cytokine treatment scRNA-seq data. **d**, UMAP of the integrated control and cytokine treatment samples, colored by sample, cell types and distance to HEOCA. **e**, Box plot comparing distance to HEOCA among control and treatment samples across different cell types. **f**, Scatter plot showing genes differentially expressed between cytokine treatment samples and the HEOCA cohorts. **g**, Dot plots displaying an example gene expression comparison of HEOCA and cytokine treatment samples across different cell types. **h**, Heatmap illustrating the DEGs GO enrichment comparison among different cytokine treatments and HEOCA cohorts. The *P* value was computed using Fisher’s exact test. **i**, Organoid perturbation expression signatures were compared for intestinal epithelial cell single-cell transcriptome data from patients with different IBDs. Box plots show the distribution of mean expression of genes differentially expressed between inflammatory or viral response conditions (compared with the HEOCA control cohort), and the overlap of DEGs between both conditions. **j**, Distribution of mean gene expression across IBD conditions for representative genes induced in viral response and inflammatory conditions. For box plots in **e**, **i** and **j**, *P* values are derived from two-tailed Mann–Whitney *U*-test. *P < 0.05, **P < 0.001, ***P < 0.001). The center represents the median; bounds show the 25% and 75% percentiles; and whiskers indicate values within 1.5× the interquartile range. Inflam, infammation.

**Extended Data Fig. 9:**
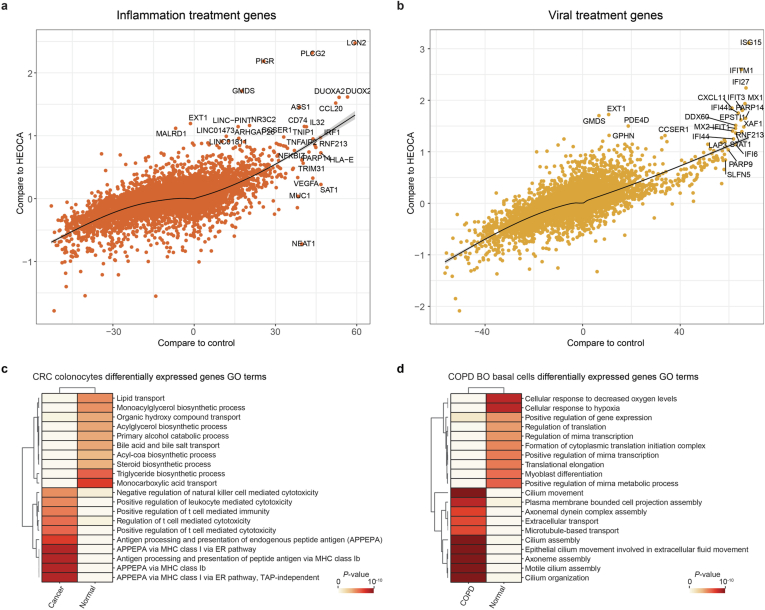
Differential gene expression comparison in perturbation samples and gene enrichment analysis in disease samples. (**a**) Scatter plot compares DEGs between inflammation perturbation/HEOCA and inflammation perturbation/control, with the top 20 genes from each comparison highlighted. (**b**) Scatter plot compares DEGs between viral perturbation/HEOCA and viral perturbation/control, with the top 20 genes from each comparison highlighted. The error bar in a,b indicated the 95% confidence interval for the regression estimate.(**c**) The top 10 enriched GO terms in differentially expressed genes between normal and colorectal cancer samples for colonocytes. (**d**) The top 10 enriched GO terms in differentially expressed genes between normal and COPD bronchial organoid (BO) samples for basal cells. The p-value was computed using Fisher's exact test.

We next sought to use the HEOCA as a cohort to assess organoid perturbations. We conducted two perturbation experiments aimed at modeling response to viral infection (interferon (IFN)α, IFNβ and IFNɣ)^34^ and acute pathogenic inflammation (TNF, Oncostatin M (OSM), IFNɣ, stem cell factor (SCF), IL-6, IL-17A and IL-18)^35–37^ (Fig. 6a). We treated ASC-derived ileum organoids with these cytokines for 24 h and performed scRNA-seq on control (4,191 cells) and treated samples from the same batch (viral response, 3,305 cells; inflammation, 2,158 cells). HEOCA projection and annotation revealed diverse cell types including stem cells, enterocytes, goblet cells and enteroendocrine cells (Fig. 6b,c). Distance-to-atlas analysis revealed that, compared with the control sample, both viral response and inflammation samples had higher distances in all cell types (Fig. 6d,e). Differential expression analysis between perturbation samples and the paired nearest neighbor cells in the HEOCA cohort revealed 618 genes specific to viral response (for example, *ISG15*, *OAS1-3*), 259 specific to inflammation (*LCN2*, *IL32*, *TNFAIP2*), 717 shared (*STAT1*, *WARS1*) and 996 genes upregulated in the atlas (Fig. 6f,g). Gene Ontology (GO) enrichment analysis showed that viral response-specific upregulated genes were enriched in functions related to the defense response to viruses, response to type I IFN and IFNβ, and regulation of autophagy. Inflammation-specific differentially expressed genes (DEGs) were associated with the inflammatory responses and cellular responses to chemokines. Genes commonly upregulated in both viral response and inflammation samples were involved in regulating epithelial cell proliferation, chromosome organization, epithelial cell migration, intracellular signal transduction and response to cytokines. By contrast, genes with higher expression in the atlas cohort were enriched in ATP biosynthetic processes, messenger RNA processing and cellular respiration (Fig. 6h). We found that DEGs identified from comparison with the HEOCA cohort were similar to the set identified through comparison with the isogenic control (Extended Data Fig. 9a,b). To assess the biological relevance of the identified states, we compared transcriptomes with counterpart epithelium in an atlas of inflammatory bowel disease (IBD) patient samples^38^. Interestingly, we found that the perturbation-induced DEGs were also differentially expressed between healthy individuals and patients with IBD (Fig. 6i, j). This finding confirms that these perturbations generate organoid cell states not prevalent in the atlas, that the integrated atlas can be used as a diverse cohort for perturbation assessment and that these perturbation states have relevance to primary counterparts.

**Fig. 7:**
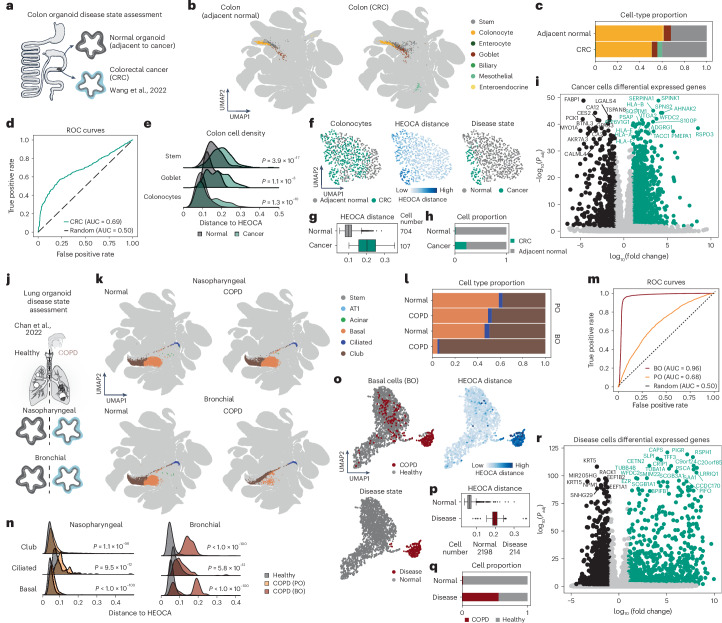
Comparison with the HEOCA healthy organoid cohort reveals disease-associated features. **a**, Overview of colorectal cancer organoid samples and their analysis through the HEOCA assessment. **b**, UMAP of adjacent normal colon and colorectal cancer samples mapped to the HEOCA colored by predicted level 2 cell type. **c**, Proportion of predicted level 2 cell type in two samples. **d**, ROC plot of cancer cell prediction using the distance to the atlas. **e**, Distance from adjacent normal and CRC cells to HEOCA is split by cell types. **f**, UMAP of CRC and adjacent normal colonocytes, colored by sample type (left), distance to HEOCA (middle) and predicted disease state (right). **g**, Box plot showing the distance to the atlas for the two disease-state clusters. **h**, Bar plot illustrates the distribution of CRC and adjacent normal cells in two distinct clusters of disease-state cells. **i**, Scatter plot showing DEGs between normal and cancer colonocytes. **j**, Overview of COPD organoid samples and their analysis through the HEOCA assessment. **k**, UMAP of normal and COPD nasopharyngeal (PO) and bronchial (BO) organoid scRNA-seq samples mapped to HEOCA, colored by predicted level 2 cell types. **l**, Proportions of predicted level 2 cell types in normal and COPD PO and BO samples. **m**, ROC plot of COPD cell prediction using distance to the atlas. **n**, Distance to HEOCA for normal and COPD PO (left) and BO (right), divided by cell types. **o**, UMAP of COPD and normal BO basal cells, colored by sample type (left), distance to HEOCA (right) and predicted disease state (bottom). **p**, Box plot presents the distance of cells to HEOCA for the two clusters of disease-state cells. **q**, Bar plot illustrates the distribution of normal and COPD BO basal cells in two distinct disease-state clusters. **r**, Scatter plot showing the DEGs between normal and COPD BO samples basal cell. For plots in **e**, **g**, **n** and **p**, *P* values are from two-tailed Mann–Whitney *U*-tests. In the box plots in **g** and **p**, the center represents the median; bounds show the 25% and 75% percentiles; and whiskers indicate values within 1.5× the interquartile range.

We next assessed the utility of the integrated atlas to understand organoid models of disease. Through comparison with the HEOCA we assess cell proportion, identify disease-associated states and perform differential expression analysis against the atlas data (Fig. 5a). We first explored colorectal cancer (CRC) using a dataset composed of CRC organoids from a patient resection and normal organoids from adjacent healthy tissue^39^ (Fig. 7a). HEOCA mapping analysis showed that CRC samples exhibited a lower percentage of mature colonocytes, and a higher proportion of stem cells (Fig. 7b,c). Interestingly, we also observed the emergence of mesothelial cells in the CRC samples, consistent with the published findings that CRC can lead to an increase in mesenchymal cells (Fig. 7b,c)^39^. Distance-to-atlas analysis distinguished cancer from normal cells, with stem cells and colonocytes showing the greatest deviation, while goblet cells remained closer to normal states (Fig. 7d,e). Subsetting and integrating colonocytes from both normal and cancer organoids identified two distinct groups: a mixed normal–cancer cluster and a cancer-specific cluster with markedly higher atlas distances (Fig. 7f–h). DEG analysis revealed higher expression levels of CRC markers such as *CEACAM6, SPINK1*, *TGFBI* and *RSPO3* in the cancer cell group (Fig. 7i). Notably, recurrent R-spondin gene fusions have been described in certain patients with CRC and this event potentiates Wnt signaling and tumorigenesis^40^. GO enrichment analysis highlighted immunity and cytotoxicity genes in cancer cells (Extended Data Fig. 9c). These analyses show the utility of distance measures to the HEOCA as a strategy to elucidate cell states that deviate healthy or otherwise normal states.

In a second assessment, we used a publicly available dataset of two different organoid types generated from cells of patients with chronic obstructive pulmonary disease (COPD) (Fig. 7j)^41^. These were derived from nasopharyngeal and bronchial stem cells of these patients respectively. Both nasopharyngeal and bronchial COPD organoids mapped to lung populations in the HEOCA, but whereas nasopharyngeal organoids resembled healthy samples, bronchial organoids exhibited an increased proportion of club cells and fewer basal cells (Fig. 7k,l). Distance-to-atlas analysis effectively distinguished normal from COPD conditions, with the bronchial COPD organoids showing notable deviations (Fig. 7m). These results matched with the original publication^41^, which showed similar differences in cell-type composition and reported differences in resistance to viral infection between the bronchial and nasopharyngeal COPD organoids. Based on atlas similarity, we observed that the nasopharyngeal normal and COPD samples showed relatively minor differences across all cell types, whereas basal cells in the bronchial COPD samples displayed a bimodal distribution (Fig. 7n). Distance to HEOCA states identified one basal cell population indicative of a disease state, which was further clarified in a heterogeneity analysis of basal cells from healthy and bronchial organoids (Fig. 7n–q). DEG analysis revealed decreased *KRT5* and *KRT15* expression, and high expression of genes known to be upregulated in COPD such as *PSCA* and *BPIFB1* (Fig. 7r). GO enrichment analysis of the DEGs shows that disease cells have enriched expression of cilium and axoneme genes (Extended Data Fig. 9d). Together these data show that the HEOCA can be used to place cell states observed in organoid disease models in a larger context, which helps to better understand holistic effects on cell composition and gene expression patterns.

**Extended Data Fig. 10:**
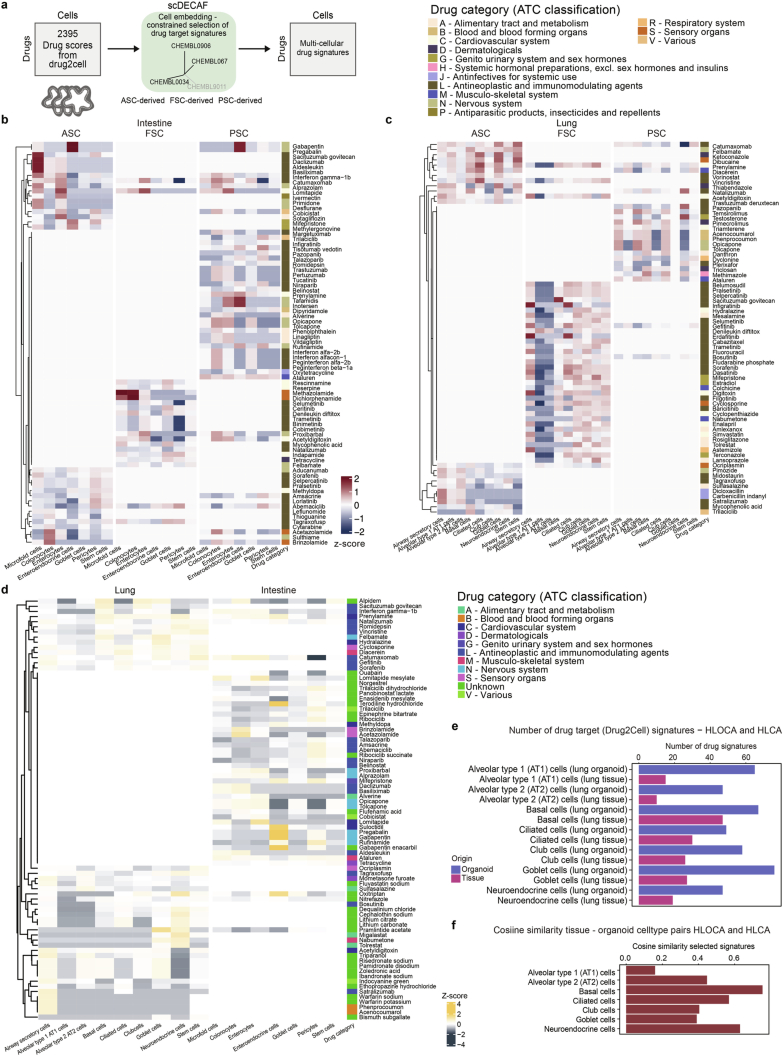
Analyzing pharmacological targets in intestinal and lung organoid cells. (**a**) Assessing the druggability similarities and differences between cell types in different organoid models by scoring drug signatures. Drug target signatures from CHEMBL database were scored using drug2cell. Heatmap of drug2cell drug signature z-scores in (**b**) intestine and (**c**) lung subsets of HEOCA with global patterns of covariation in more than two cell types (aka multicellular drug target signatures) identified by scDECAF method for drug2cell, for ASC-derived, FSC-derived and PSC-derived organoid models. Drugs are annotated by Anatomical Therapeutic Chemical (ATC) classification. Drugs with unknown ATC categories are not shown. (**d**) Cell-of-origin agnostic comparison of drug target signatures between lung and intestine organoids in common and uncommon cell types between the two tissue types. Heatmap of z-scores of multicellular drug signatures identified in lung and intestine. Drugs are annotated by ATC classification. (**e-f**) Assessing the viability of organoid cell types for drug screening in primary tissue by comparing multicellular drug target signatures between lung organoid (HLOCA) and lung primary tissue (HLCA). (**e**) Number of drug signatures found per cell type in lung organoid and primary tissue. (**f**) Cosine similarity of multicellular drug target signatures for primary tissue-organoid cell type pairs.

Finally, we provide an assessment of the viability of organoids derived from different source types (PSC, FSC, ASC) for drug target screening. We used Drug2Cell (D2C)^42^ to score the expression levels of 2,395 drug target signatures from the CHEMBL database in single cells from HEOCA, and used scDECAF^43^ to select drug target signatures that exhibited global covariation in two or more cell types to identify multicellular drug signatures (Extended Data Fig. 10a and Supplementary Table 6). Comparison between ASC-, FSC- and PSC-derived intestine and lung organoid models showed substantial differences in drug target pathway activities (Extended Data Fig. 10b,c). Druggable targets in categories including alimentary tract metabolism, systemic hormones, anti-infectives, antiparasitics, and antineoplastic and immunomodulating agents were implicated in signatures that varied between cell types and stem cell sources. Comparison between lung and intestine organoid models across all cell types suggested that many druggable targets are distinct between the two tissues and cell types common to both intestine and lung (for example, stem and goblet cells) have unique features (Extended Data Fig. 10d–f).

In summary, our analyses demonstrate that HEOCA is a technically and biologically diverse cohort that can be leveraged to evaluate organoid models, identify pathways impacted by perturbations, and, more broadly, explore the ontogeny of human biology.

## Discussion

Single-cell transcriptome sequencing technologies have advanced organoid research by offering a powerful set of experimental and computational tools to investigate cell types present in these complex 3D models. Despite immense progress, it remains a challenge to understand and quantify organoid fidelity and to place variation between organoid datasets into a larger context. To begin to address these challenges, we have built an integrated cell atlas of organoids that model endoderm-derived tissues, incorporating organoid datasets that have been generated from multiple different types of stem cells and protocols. We have established a framework for integration and harmonized cell-type annotation, which makes interpreting cell heterogeneity between organoid datasets tractable. Harmonization of cell-type annotation and nomenclature is challenging, and we envision that comprehensive integrated reference atlases across the human lifespan will enhance the robustness of cell annotation in organoid datasets. With regard to atlas building, single-cell transcriptome data from diverse experimental designs can introduce strong technical noise because of batch effects, protocol variation, genomic method and other technical biases, making data integration challenging. To overcome this, we evaluated existing integration methods and identified a suitable model based on bioconservation and integration metrics. This integration method, scPoli, is structured to incorporate additional data, enabling rapid comparison of datasets through the sc2heoca package or ArchMap website (https://www.archmap.bio). We find that there is notable variation in organoid cell composition, prevalence of off-target cells and overall cell state similarity. This variation, and comparison with available reference atlases, revealed that current organoid technologies cover a large diversity of human cell types and states, and particularly that organoids can model both early stages of fetal development as well as stages of adulthood. This result helps to clarify the use of human organoid technologies to explore development, model disease and test therapeutics.

Through cross-organ, multiorganoid integration, it was possible to identify off-target cells, a particular problem in PSC-derived organoids because of incomplete specification, as well as to distinguish cell states that markedly differed from states present in the atlas. This ability to distinguish nonpresent states is helpful to assess protocols, as well as to identify features of disease models that are absent in normal, healthy organoids. Indeed, the integrated HEOCA presents an opportunity to place an endodermal organoid dataset into a relationship with datasets generated from a technically and biologically diverse cohort. There is still a major challenge with organoid fidelity quantification, particularly with rare cell types or transient ontogenetic states, because there is not yet a complete and integrated atlas of human cell-type diversity during development and adulthood from primary tissues. Comprehensive integrated reference atlases across the human lifespan, in health and disease, together with diverse organoid models in normal and perturbed conditions, will help to clarify the full potential of the human genome. Altogether, the HEOCA will serve as a valuable resource for the organoid research community and a foundation to expand the ability to model human biology.

## Methods

The experiments conducted in this study did not require approval from a specific ethics board.

### Statistics and reproducibility

To integrate the atlas, all available datasets were included, with no sample exclusion. For integration method comparisons and sample variance effect analyses, random samples were selected from the full dataset. Reproducibility codes for the analyses are available online via GitHub, as detailed in the ‘Code availability’ section. All statistical methods are described in the corresponding sections of the paper.

### Organoid culture, cytokine treatment and scRNA-seq

Human intestinal tissue samples were obtained and experimental procedures performed within the framework of the nonprofit foundation HTCR (Munich, Germany) including informed patient consent. Ileal organoids were derived and maintained according to previously published culture conditions^10^. For the cytokine treatments, organoids were dissociated into five- to ten-cell fragments using TrypLE (Invitrogen) and reseeded in Matrigel. After 6 days, organoids were treated for 6 days by supplementing the culture medium with 50 ng ml^−1^ TNF and 200 ng ml^−1^ RANKL (Acro Biosystems) for TNF treatment^28^ or with 400 ng ml^−1^ IL-4 and 40 ng ml^−1^ IL-13 (Acro Biosystems) for IL-13 and IL-4 treatment. To model host cell responses to viral infection, organoids were treated for 1 day with 1 ng ml^−1^ IFNα, 1 ng ml^−1^ IFNγ (Acro Biosystems) and 5 ng ml^−1^ IFNβ (PeproTech)^34^. To model acute pathogenic inflammation, organoids were treated for 1 day with 10 ng ml^−1^ TNF, 10 ng ml^−1^ IL-6, 500 ng ml^−1^ IL-17A, 1 ng ml^−1^ IFNγ (Acro Biosystems), 50 ng ml^−1^ IL-18, 100 ng ml^−1^ OSM (BioLegend) and 100 ng ml^−1^ SCF (MedChemExpress)^35–37^. After the indicated treatment durations, organoids were dissociated for scRNA-seq using the Neural Tissue Dissociation Kit (P) (Miltenyi Biotec) as described previously^23^. First, culture medium was removed and organoids were incubated in Cell Recovery Solution (Corning) for 40 min at 4 °C. Next, organoids were transferred to 1% bovine serum albumin (BSA)-coated tubes using HBSS–1% BSA buffer while pipetting thoroughly to fragmentize the organoids. Organoid fragments were centrifuged at 500*g*, 5 min, 4 °C. Each cell pellet was resuspended in prewarmed buffer X mixed with 25 μl of enzyme P. Cells were incubated for 15 min at 37 °C combined with mechanical dissociation by pipetting every 5 min. Next, 5 μl of enzyme A in 10 μl of buffer Y was added to the digest and incubated for a further 10 min combined with pipetting every 5 min. Cells were subsequently washed twice with HBSS–1% BSA buffer and filtered through a 40-μm filter coated with 1% BSA. Single cells were counted using a Countess 3 FL Automated Cell Counter (Invitrogen) and kept on ice. Dilutions of ~1,000 cells per μl in 50–60 μl of HBSS–1% BSA buffer were prepared and immediately processed using the 10x Chromium Next GEM Single Cell 3′ Reagent Kit (v.3.1) according to the manufacturer’s instructions. Libraries were sequenced on Illumina’s NovaSeq6000.

### Data collection

The scRNA-seq data used in this study were obtained from the original papers (Supplementary Table 1). If the raw fastq files were available, they were downloaded. The seq2science (v.1.2.2)^44^ method was used to download the raw fastq files from the Gene Expression Omnibus database (https://www.ncbi.nlm.nih.gov/geo/) or BioStudies database (https://www.ebi.ac.uk/biostudies/). The reads were aligned to the GRCh38 genome and Ensembl 98 gene annotation using STARsolo (STAR v.2.7.10b)^45^. In cases in which the raw FASTQ files were not available, the raw counts were downloaded instead. The downloaded counts and the counts obtained from the realigned reads were merged for subsequent analysis.

### Data normalization

To integrate the data, we combined the count data from all the samples into a unified dataset. For subsequent analysis, we retained only the genes classified as protein-coding genes and long noncoding RNA genes. The low-quality cells in each sample were filed. The raw counts were then normalized to a total count of 10,000 and log-transformed. Given these normalized counts, the top 3,000 highly variable genes were identified using the default settings in Scanpy. These highly variable genes were selected for further downstream analysis.

### Cell-type annotation

Cell-type annotation was performed using the snapseed method (https://github.com/devsystemslab/snapseed). For each sample, the raw counts were normalized to a total count of 10,000 and then log-transformed. From these normalized counts, the top 3,000 highly variable genes were identified using the default settings in Scanpy. These highly variable genes were selected as the subset for further downstream analysis. Principal component analysis (PCA) was performed on the normalized data, and the top 30 principal components were chosen for calculating the *k* nearest neighbors (*k*NN). Using the *k*NN, a UMAP was generated to visualize the data in a lower-dimensional space. To cluster the data, the Leiden clustering method with a resolution of 2 was applied. This clustering approach helped to identify distinct groups of cells based on their gene expression patterns. Previously defined marker genes associated with specific cell types were used to guide the annotation process. To annotate cell types in each cluster, the snapseed method was used. This method calculates the area under the receiver operating characteristic (ROC) curve (AUC) and fold change values for each marker gene in relation to the cluster. If multiple markers were available for a particular cell type, the maximum AUC and fold change values were selected. The average AUC and fold change values were used to represent the specific cell type, and the most specific cell type was annotated for each cluster based on these criteria.

### Pseudo-bulk analysis

For gene expression level, we merged all counts in each organoid sample by genes using the adpbulk^46^ method and applied a natural logarithm transformation to one plus the counts. We then selected the top 500 highly variable genes and calculated PCA based on their expression. From principal components 1 and 2 (PC1 and PC2), we selected the top 200 and bottom 200 loading genes for GO enrichment analysis using the GSEApy^47^ method.

For the scPoli embedding level, we calculated the mean scPoli embedding for each organoid sample using the adpbulk^46^ method, followed by PCA based on the mean embedding. A linear model was then used to calculate the covariance between principal components and sample counts, stem cell source, scRNA-seq method, tissue type and publication.

### Data integration benchmarking

To benchmark and compare different integration methods, we selected ten random samples from the dataset for validation, repeating this process ten times. Twelve integration methods, including PCA, Seurat (v.3, v.4 and v.5), scVI, scANVI, scPoli, bbknn, harmony, combat, CSS (pearson) and CSS (spearman)^14–21^ were applied to the data to assess their performance in integrating the samples. The scIB method, a benchmarking tool, was used to evaluate and compare the results obtained from these integration methods. In the scPoli model, we configured the following parameters for effective training and integration: embedding_dim was set to 3; hidden_layer_sizes were determined as the square root of the total number of cells. During the training phase, we used the following settings: early_stopping_metric was set to val_prototype_loss; mode was set to min; threshold was set to 0; patience was set to 20; reduce_lr was enabled, with lr_patience set to 13 and lr_factor set to 0.1; n_epochs were set to 5; pretraining_epochs were set to 4; eta was set to 10; alpha_epoch_anneal was set to 100.

### Sample variances across integration benchmark

To benchmark and compare how different sample variances affect integration, we selected ten random samples from the dataset as a control and another ten random samples with the same sample variance, such as samples from the same organoid tissue type. Each group of selected samples was integrated using the scPoli method with the same settings as in the HEOCA integration. The scIB method was then used to benchmark the different integrations. The difference in scIB output between the control and each sample variance pair was calculated to represent the effect of sample variance on integration. For benchmarking the effect of cell number variance, we selected the same ten samples as the control and performed a random subset of each sample to the median or mean number of cells in all the HEOCA samples. The subsequent comparison followed the same procedure as the other sample variance benchmarks.

### Cell-type reannotation

After integration, we recluster all cells in the atlas based on the scPoli integrated embedding using the Leiden method with a resolution of 10 (HEOCA and HIOCA) and 10 (HLOCA), respectively. Annotations were then assigned to each cluster using the dominant cell type per cluster. Some clusters of cells were adjusted according to the marker genes expression.

### Marker gene refinement

We randomly subset 100,000 cells from the atlas. For each cell type, we used the Wilcoxon rank-sum test to identify DEGs, selecting the top ten genes as marker genes for each cell type. We combined the selected marker genes and performed hierarchical clustering on the resulting gene set.

### Cross-organ primary tissue integration

The human fetal endoderm tissue atlas was downloaded^23^. The normal endoderm tissues including the esophagus, lung, liver, intestine, stomach and pancreas were subsetted. The top 3,000 highly variable genes were subsetted for data integration. The cells in each tissue were integrated using the scPoli method^20^, with the cell_type serving as the cell-type key for integration and with the same parameters used in the HEOCA integration. The scPoli model was saved for the downstream comparison. The Tabula Sapiens multiple-organ adult single-cell transcriptomic atlas of humans was downloaded (https://tabula-sapiens-portal.ds.czbiohub.org/)^6^. The endoderm tissues including the liver, lung, pancreas, small intestine, large intestine, prostate and stomach were subsetted. The endothelial, epithelial and stromal compartments of cells were subsetted. The top 3,000 highly variable genes were subsetted for data integration. The cells in each tissue were integrated using the scPoli method^20^, with the cell_ontology_class serving as the cell-type key for integration and with the same parameters used in the HEOCA atlas integration. The scPoli model was saved for the downstream comparison.

### Organoid off-target analysis

For each organoid sample, the same set of variable genes used in the primary tissue atlas (adult or fetal) was chosen, and the scPoli query was executed using identical parameters to those used in the primary tissue atlas training model. The UMAP embedding was transformed using the primary tissue atlas UMAP model. For each cell, the system selected its 100 nearest neighbors from the HEOCA dataset. The predicted tissue for the cell was assigned based on the tissue that was most frequently observed among its 100 nearest neighbors.

### Correlation to primary tissue

To compare and correlate cell states in primary tissue and organoid models, the miloR^48^ method was used to define and construct neighborhood graphs for each data source separately. We computed the transcriptional similarity graph for the primary tissue reference using 30 nearest neighbors and the UMAP representation of latent representations of integrated primary tissue cells. To compute the transcriptional similarity graph for the organoid reference, we used the 30 nearest neighbors and the UMAP representation of integrated embedding of organoid cells. Single-cell organoid data were integrated using scPoli and 3,000 highly variable genes as described earlier. We used the default parameters for all the remaining computational steps in building the neighborhood graphs. We then used the R package scrabbitr^24^ to compute the correlation between each pair of neighborhoods in the primary tissue and organoid reference and to annotate the results at cell-type or tissue level. The neighborhood correlations were computed using 3,000 highly variable genes that were found in the highly variable genes in the primary tissue single-cell reference atlases. This step results in two neighborhood correlation matrices: a primary tissue-correlation matrix in which each entry marks correlation of the expression profile of a given neighborhood in the primary tissue with the HEOCA, and an organoid-correlation matrix that stores the correlation of expression profiles in each neighborhood of the organoid atlas with the primary tissue atlas. This procedure was also repeated for each organoid derivation protocol, that is ASC-, FSC- and PSC-derived protocols. To compare the correlation between cell states in the primary tissue and between organoid derivation protocols, we subtracted the primary tissue–neighborhood correlation matrices computed with respect to neighborhoods for each derivation protocol. This approach of comparing primary tissue and organoid by correlation of neighborhood graphs is more reliable than the alternative reference mapping strategy, because it removes the dependance of the reliability and accuracy of the conclusions to mapping uncertainty, and allows for computing correlation statistics on graphs that are constructed based on transcriptional similarity of cells in each data source.

### Velocity and pseudotime analysis

For RNA velocity analysis of the HIOCA, we first excluded samples missing splicing information. We then applied scVelo^49^ to generate a UMAP representation with stream trajectory visualization. The velocity pseudotime, spanning from stem cells to enterocytes and colonocytes, has been rescaled to a range of 0 to 1. We calculated and displayed the average expression of markers in specific bins.

### Intestine organoid atlas integration

The top 3,000 highly variable genes were subsetted for data integration. To integrate all the cells, we applied the scPoli method with the same parameters used in the HEOCA atlas integration. The scPoli model was saved for the downstream comparison.

### Lung organoid atlas integration

Lung organoid single-cell data curated from different studies was subsetted on top 3,000 highly variable genes for integration. We applied scPoli to learn 30-dimensional latent representations of the cells, and 10-dimensional latent representations of the samples using a neural network with 2 hidden layers each of size 512. The network was trained setting n_epochs=12, pretraining epochs to 10, eta=10, patience=20, lr_patience=13, lr_factor=0.1, alpha_epoch_anneal=100, reduced_lr=True and prototypical loss of the validation set as the early stopping criteria. The scPoli model was saved for the downstream comparison.

### Intestine primary tissue atlas integration and compression of organoid samples

The scRNA-seq data from both duodenum fetal and adult primary tissues were obtained from two research papers^23,25^. We focused on epithelial cells and subsetted them for analysis. The top 3,000 highly variable genes were subsetted for data integration. To integrate all the cells, we applied the scPoli method with the same parameters used in the HEOCA atlas integration. The scPoli model was saved for the downstream comparison. For each organoid sample, the same set of variable genes used in the primary tissue atlas was chosen, and the scPoli query was executed using identical parameters to those used in the primary tissue atlas training model. The UMAP embedding was transformed using the primary tissue atlas UMAP model. For each cell, the system selected its 100 nearest neighbors from the primary tissue dataset. The predicted cell type for the cell was assigned based on the tissue that was most frequently observed among its 100 nearest neighbors. To identify DEGs in primary tissue stem cells and enterocytes, we subsetted these cell types and used a linear model to calculate the covariance between sample age and gene expression for each gene. The top 100 genes with the highest coefficients were selected as DEGs. The GSEApy method was then applied to identify the top GO-enriched terms associated with these genes.

To identify the heterogeneity of intestinal organoid stem cells and enterocytes, cells from the HIOCA were subsetted. Integration was performed using the CSS method^18^ based on 1,000 highly variable genes across all cells. Leiden clustering with a resolution of 0.1 was applied to identify subclusters. The Wilcoxon rank-sum test was used to identify DEGs among subclusters, and GO enrichment analysis was conducted on the top 500 DEGs of each group using GSEApy.

### Lung primary tissue atlas integration and compression of organoid samples

The scRNA-seq data from both duodenum fetal and adult primary tissues were obtained from two research papers^6,23^. The top 3,000 highly variable genes were subsetted for data integration. To integrate all the cells, we applied the scPoli method with the same parameters used in the HEOCA atlas integration. The scPoli model was saved for the downstream comparison. For each organoid sample, the same set of variable genes used in the primary tissue atlas was chosen, and the scPoli query was executed using identical parameters to those used in the primary tissue atlas training model. The UMAP embedding was transformed using the primary tissue atlas UMAP model. For each cell, the system selected its 100 nearest neighbors from the primary tissue dataset. The predicted cell type for the cell was assigned based on the tissue that was most frequently observed among its 100 nearest neighbors.

### Dataset incorporation

Samples of scRNA-seq raw reads were mapped to the human genome, and counts of the matrix were obtained. The same set of variable genes used in HEOCA was chosen, and the scPoli^20^ query was executed using identical parameters to those used in the HEOCA training model. The UMAP embedding was transformed using the HEOCA UMAP model. For each cell, the system selected its 100 nearest neighbors from the HEOCA dataset. The predicted cell type for the cell was determined by assigning it the cell type that was most frequently observed among its 100 nearest neighbors at the level 2 cell-type classification. Similarly, the predicted tissue for the cell was assigned based on the tissue that was most frequently observed among its 100 nearest neighbors.

### Reconstruction of matched sample reference in HEOCA

For each cell in the organoid protocols, organoid perturbation, and disease samples, a matched HEOCA cell was reconstructed using the top ten *k*NN in HEOCA. The mean expression of these ten neighbors was calculated to represent the expression profile of the matched sample reference in HEOCA. In addition, the mean *k*NN distance of these ten neighbors was used to represent the cell’s distance to the HEOCA.

### *F* test-based differential expression analysis between a sample and HEOCA

To compare expression levels of the samples, the above-mentioned matched sample reference in HEOCA was identified. The expression difference per gene for each cell pair was calculated based on the log-normalized expression values. For each gene, the variance over the calculated expression difference per cell pair was compared with the sum of squared expression differences normalized by the number of cell pairs. An *F* test was applied to test for differential expression for each gene.

### Organoid cytokines treatment analysis

scRNA-seq reads for each sample were mapped to the human genome, and gene counts were generated using CellRanger. These counts served as input for sc2heoca, with default settings used to map all samples to HEOCA. During mapping, each cell was annotated with a level 2 cell type, and the distance to HEOCA was calculated. Cell proportions were determined based on the mapping annotations. For the raw integration of perturbation samples, three samples were merged, highly variable genes were identified using Scanpy with default settings, and the samples were integrated using the ComBat method. The sc2heoca package with default settings was used to identify DEGs, and GO enrichment analysis was performed using GSEApy on the DEGs of each group. DEGs between each treatment sample and control sample were calculated using the Wilcoxon rank-sum test in Scanpy (v.1.9.3).

The scIBD database^38^ was downloaded, and samples from healthy individuals, and patients with colitis, Crohn’s disease and ulcerative colitis were extracted. Only epithelial cells were selected for downstream analysis. Pseudo-bulk gene expression was calculated for each individual, and the DEGs identified in the previous step were subsetted. The mean expression of these genes across patients was used to compare gene expression between inflammatory and viral response conditions.

### Disease sample analysis

The disease sample analysis is similar to the sample incorporation step. The raw count matrices were downloaded from the original papers. The same set of variable genes used in HEOCA was chosen, and the scPoli^20^ query was executed using identical parameters to those used in the HEOCA training model. The UMAP embedding was transformed using the HEOCA UMAP model. For each cell, the system selected its ten nearest neighbors from the HEOCA dataset. The predicted cell type for the cell was determined by assigning it the cell type that was most frequently observed among its ten nearest neighbors at the level 2 cell-type classification. The predicted tissue for the cell was assigned based on the tissue that was most frequently observed among its ten nearest neighbors. For each cell, the mean distance of its ten nearest neighbors was assigned as its mean distance to HEOCA.

In the analysis of DEGs between colon cancer organoid colonocytes and bronchial COPD organoid basal cells, we performed separate subsetting for all colonocytes and basal cells. For each dataset, we isolated the top 3,000 highly variable genes. We then integrated these subsets of cells using the bbknn method^14^. To cluster the two datasets, we applied the Leiden method with resolutions of 1 and 2 in two datasets. The clusters predominantly associated with the disease were selected as disease state cells, while the remaining clusters were categorized as normal state cells.

### Drug target analysis

We used D2C^42^ to score the expression levels of 2,395 drug target signatures in single cells from the human organoid cell atlas and the human lung cell atlas. D2C scores were scaled to mitigate scale differences between different datasets in the atlas. We used the R package scDECAF (v.0.99.0)^43^ to select drug target signatures that exhibited global covariation in one or more cell types in HEOCA and HLCA primary tissue atlases. The inputs to scDECAF were the scaled D2C *z*-scores and the cell embeddings from the atlases. The shrinkage operator in scDECAF was set to lambda = exp(−1.3) based on reconstruction error plots made available in the scDECAF package. We assigned a drug signature to a cell type if more than 50% of the cells from the cell type had a signature score above median across all cell types. Multicellular drug target signatures were identified whether a drug signature was selected in at least two cell types. To assess druggability potential of organoid cell types, we computed the cosine similarity for cell-type pairs in organoid and primary tissue based on multicellular drug signatures identified in primary tissue and organoid models.

### Reporting summary

Further information on research design is available in the Nature Portfolio Reporting Summary linked to this article.

## Online content

Any methods, additional references, Nature Portfolio reporting summaries, source data, extended data, supplementary information, acknowledgements, peer review information; details of author contributions and competing interests; and statements of data and code availability are available at 10.1038/s41588-025-02182-6.

## Supplementary information

Supplementary Information Supplementary methods and references.

Reporting Summary

Supplementary Tables Supplementary Tables 1–6.

## Data Availability

The HEOCA (raw and normalized counts, integrated embedding, cell type annotations and technical metadata) is publicly available and can be downloaded at CELLxGENE (https://cellxgene.cziscience.com/collections/b4d13dc2-9b75-401d-9d9a-6d1468c17d90), the Cell Annotation Platform (CAP) (https://celltype.info/project/604) and via Zenodo at 10.5281/zenodo.8181495 (ref. ^50^). The HEOCA core reference model and embedding for the mapping of new data to the HEOCA and human intestinal organoid cell atlas can be found via Zenodo at 10.5281/zenodo.8181495 (ref. ^50^). The GRCh38 genome assembly can be found at https://www.ncbi.nlm.nih.gov/datasets/genome/GCF_000001405.26/. The scRNA-seq data of intestine organoid generated in this study have been deposited in the Gene Expression Omnibus (GEO) database under the accession number GSE287233.

## Code availability

Code for scRNA-seq cell type annotation is available as a Python package, deposited at 10.5281/zenodo.15075590 (ref. ^51^) and maintained via GitHub at https://github.com/devsystemslab/snapseed. Code for mapping protocol data and disease data to HEOCA is available as a Python package via Zenodo at 10.5281/zenodo.15075673 (ref. ^52^) and maintained at GitHub (https://github.com/devsystemslab/sc2heoca), code for all the analysis in this paper is available via Zenodo at 10.5281/zenodo.15075693 (ref. ^53^) and via GitHub at https://github.com/devsystemslab/HEOCA.
