# Supplementary material for: An integrated transcriptomic cell atlas of human endoderm-derived organoids
Source: Nat Genet. 2025 May 12;57(5):1201–12. doi: 10.1038/s41588-025-02182-6 (PMC12081310; doi:10.1038/s41588-025-02182-6)
Supplement: Supplementary file 1 — Supplementary methods and references. [file 41588_2025_2182_MOESM1_ESM.pdf]

nature genetics

Supplementary information https://doi.org/10.1038/s41588-025-02182-6

An integrated transcriptomic cell atlas of human endoderm-derived organoids

In the format provided by the authors and unedited

Supplementary Methods

Generation and culture of PSC-derived human intestinal organoids

All PSC lines were cultured with mTESR/mTESR-plus (STEMCELL Technologies 85850/ 100-0276) media on Matrigel (Corning, 354277) coated plates. The cells were passaged when they reached 80-90% confluency every 4-5 days. PSCs were differentiated into HIO following the previously described protocols[1–3]. In brief, PSCs were induced into definitive endoderm by using 100 ng/ml Activin A (R&D, 338-AC) in RPMI-1640 for three days with increasing concentrations of FBS (0%, 0.2%, and 2%). Then, midgut/hindgut patterning of endoderm was directed with 500 ng/ml FGF4 (Peprotech, 100-31) and 2uM CHIR99021 (Stem Cell Technologies, 72052) with daily media changes for up to 6 days in the presence of 2% fetal bovine serum (FBS). Spheroids were collected after 96, 120, and 144 hours of patterning and embedded in Matrigel (Corning, 354234/356231), cultured in ENR media (mini gut basal media supplemented with 100 ng/ml EGF (R&D, 236-EG), 100 ng/ml purified Noggin-Fc[4] and R-Spondin-1-Fc (5% conditioned media)[5]. The mini gut basal media consists of DMEM/F-12, HEPES (Thermo Fisher Scientific, 11330032), and 1x B27 supplement (Thermo Scientific, 12587001). All media used in the differentiation process contain 1x Penicillin-Streptomycin (Thermo Scientific, 15140122). Organoid media was changed every 3 days. HIOs were split once the mesenchyme outgrew Matrigel, and excessive cell debris accumulated in the core every 7-10 days.

For the suspension HIO protocol[6], the modifications to the original protocol described above is that the budded spheroids were collected and cultured in ultra-low attachment plates without splitting to protect the mesothelial layer. During organoid growth, Noggin and R-spondin were removed after 3 days of growth.

Another exception to the main protocol was HIO-ECs (vHIO)[7] grown with minor adjustments to introduce endothelial cells co-development with a first three days of spheroid growth with minigut media containing EGF (100 ng/ml), VEGF (50 ng/ml), bFGF (25 ng/ml), and BMP4 (25 ng/ml) that followed by supplementation with EGF (100 ng/ml) and VEGF (25 ng/ml) for the rest of the *in vitro* growth of the organoids as described previously.

Human intestinal organoid transplantation

HIOs were cultured in Matrigel domes in ENR media for a period of 4 weeks until transplantation. All the HIOs were grown in ENR media *in vitro* except the iPSC72.3- and two H9-derived HIOs which were grown with ENR media till day 3 and then changed to EGF media[7] and passaged weekly. On the day of transplantation, HIOs were mechanically dissociated from the Matrigel and implanted beneath the kidney capsules of immunocompromised NOD-SCID IL2Rg null (NSG) mice (Jackson Laboratory, strain number 0005557), following established protocols[8,9]. Briefly, mice were anesthetized using 2% isoflurane, and a left-flank incision was made to expose the kidney after proper shaving and sterilization with isopropyl alcohol. HIOs were implanted beneath the mouse kidney capsules using forceps. Prior to closure, an

intraperitoneal flush of Zosyn (100 mg/kg; Pfizer) was administered. Mice were euthanized after 8 weeks for scRNA-seq. For particular experiments, location of transplantation was changed to mesentery[10].

Mice housing rooms employ centrally controlled and monitored light cycles that utilize a 12-hour light / 12-hour dark photoperiod. Temperatures are maintained within plus or minus 2 degrees throughout a range of ~18-26°C with 30-70% humidity. Experiments were performed in compliance with and approved by the Institutional Animal Care and Use Committee (Protocol # PRO00006609).

Media compositions for human enteroids

To support growth and maintenance of human ASC-derived intestinal organoids in 3D we culture organoids in Matrigel domes, WERN-complete media was used[11–13]. The composition of the complete media: 50% L-WRN Condition Medium (University of Michigan), GlutaMAX Supplement (2 mM, Thermo Fisher Scientific, Cat# 35050061), HEPES (10 mM, Fisher, Cat# 15630080), Primocin (100 µg/mL, Invivogen, Cat# ant-pm-1), N-2 supplement (1×, Thermo Fisher Scientific, Cat# 17502048), B-27 supplement (1×, Thermo Fisher Scientific, 17504044), N-Acetyl-L-Cysteine (1 mM, Sigma-Aldrich, A9165), Recombinant Human EGF Protein, CF (100 ng/mL, R&D Systems, 236-EG-01M), A 83-01 (500 nM, Stem Cell Tech, 72022), SB 202190 (100 µM, Stem Cell Technologies, 72632), Nicotinamide (10 nM, Sigma-Aldrich, Cat# N5535), and Advanced DMEM/F-12 (Thermo Fisher Scientific, Cat# 12634028).

The composition of the budding differentiation media for human enteroids adapted[14] with minor modifications : 50% L-WRN Condition Medium (University of Michigan), GlutaMAX Supplement (2 mM, Thermo Fisher Scientific, Cat# 35050061), HEPES (10 mM, Fisher, Cat# 15630080), Primocin (100 µg/mL, Invivogen, Cat# ant-pm-1), N-2 supplement (1×, Thermo Fisher Scientific, Cat# 17502048), B-27 supplement (1×, Thermo Fisher Scientific, 17504044), N-Acetyl-L-Cysteine (1 mM, Sigma-Aldrich, A9165), IGF1 (100 ng/mL, BioLegend, Cat# 590904) , FGF-basic (50, ng/mLPeprotech, Cat# 100-18B) , and A 83-01 (500 nM, Stem Cell Tech, 72022), Nicotinamide (10 nM, Sigma-Aldrich, Cat# N5535), and Advanced DMEM/F-12 (Thermo Fisher Scientific, Cat# 12634028).

Single-cell dissociation of organoids and tissues

PSC-derived human organoids, tHIOs and ASC-derived enteroids dissociated to single cells using the previously established protocol[15]. Briefly, to prevent cell adhesion, all tubes and pipette tips were coated with 1% BSA in HBSS. The organoids were mechanically dislodged, pipetted up and down to remove excess Matrigel, and pooled in a Petri dish. After removing excess media, the organoids were minced into smaller fragments in HBSS. The tissue pieces were transferred to a 5 ml conical tube containing Mix 1 from the Neural Tissue Dissociation Kit (Miltenyi Biotec, 130-092-628) and incubated on a shaker for 15 minutes at room temperature. Mix 2 was added to the tube, and every 10 minutes for another 20-30 minutes, the mix was agitated with a P1000 pipette tip. The cells were then filtered through a 70 µm strainer and washed

three times at 400g for 5 minutes with 1% BSA in HBSS. Cell counting was performed using Countess and the process was immediately continued with 10x Chromium for single-cell droplet generation. The libraries were prepared using the NextGEM Single Cell 3' v3.1 kit according to the manufacturer's instructions. The libraries were sequenced on Illumina's Novaseq6000 system. For detailed information on each library and associated metadata refer to the Supplementary Table.

Human liver organoid

Human fetal liver tissue (16 gestational week) was obtained following elective pregnancy termination and informed written maternal consents from Cercle Allocation Services, USA. Tissue dissection and organoid culture were performed as described previously[16]. The organoid culture at the moment of characterization contained a mixture of phenotypic hepatocyte- and cholangiocyte-enriched organoids.

Human stomach organoid

Human stomach tissue was obtained and experimental procedures performed within the framework of the non-profit foundation HTCR (Munich, Germany) including informed patient's consent. Healthy tissue was obtained as part of resections on gastric cancer, and both antrum and pylorus organoids were established from the same donor. Tissue dissection and organoid culture were performed as described previously[17].

Time course colon organoids

Mature organoids are collected by rinsing the maintenance well using 1-2 ml cold DMEM/F12 (Stem Cell Technologies) + 15 mM HEPES + 1x GlutaMax (1:100 #35050-038) + 100 µg/ml PenStrep (#15140-122) (=DMEM+++) and transferring the solution into a 15 ml falcon tube. Next, DMEM+++ is added until a total volume of 7 ml is reached before centrifuging the tube at 400g for 4 min at 4°C. Supernatant is discarded and organoids are resuspended into 2 ml of prewarmed 0.05% Trypsin + EDTA (gibco, 25300- 054) and incubated for ~10 min at 37°C. Trypsinization is stopped using 2 ml of a trypsin-inhibitor (Thermo Fisher Scientific, 17075029) as well as 3 ml of DMEM+++ after reaching a single-cell suspension. Singularized organoids are then centrifuged at 600g for 5 min at 4°C and supernatant is discarded subsequently. Next, the cell pellet is resuspended into START medium and then filtered through a 40 µm filter to remove large debris and cell clumps. Optional: FAC-sorting for single cells to remove remaining smaller debris as well as doublets. Cells are then counted before being further processed. Finally, the cell-suspension is diluted to 25-50 cells/µL into a ~60% Matrigel : ~40% START medium mixture (we have tested dilutions with a Matrigel proportion of 50 to 70%). Here, lower seeding densities between 25 and 50 cells per µL are recommended as very dense plating affects the morphology and growth of organoids negatively – this needs to be optimized for every line. For maintenance, the Matrigel mixture is plated into cell culture plates in 5-10 µL droplets and are covered after a 30 min 37°C solidification period with warm START medium. Medium is changed after 4 days to BALANCE, and is refreshed once with new BALANCE medium at day

7 until maturation is reached, typically at day 10. START and BALANCE media compositions are described in Oost et al., 2023 (currently in revision and will be made available).

Mini-colon organoid

Human colon organoids were maintained for 4 days starting from mechanically dissociated fragments in Matrigel (Corning, 356231) or Matrigel supplemented with 20% Collagen I (Advanced Biomatrix, 5225) in Advanced DMEM/F12 with penicillin/streptomycin, 1× Glutamax and 10 mM HEPES, supplemented with 1× B27 supplement (Gibco), 1 µM N-acetylcysteine (Sigma-Aldrich), 0.5 nM Wnt Surrogate-Fc Fusion Protein (U-Protein Express B.V., N001), 100 ng/ml Noggin (EPFL Protein Expression Core Facility), 500 ng/ml R-Spondin 1 (EPFL Protein Expression Core Facility), 100 ng/ml recombinant human IGF-1 (BioLegend), 50 ng/ml recombinant human FGF-2 (Peprotech), 10 nM gastrin, 100 ng/ml recombinant human NRG1 (R&D, 5898-NR) and 500 nM A83-01 (Tocris). Human mini-colons were maintained for 14 and 21 days, where starting from day 7 apical medium was switched to 'ENR' composed of 50 ng/ml recombinant human EGF (R&D, 236-EG), 100 ng/ml Noggin (EPFL Protein Expression Core Facility), 500 ng/ml R-Spondin 1 (EPFL Protein Expression Core Facility), and organoid medium as described above was maintained on the basal side.

Human mini-colons cultured in biological triplicates were extracted from the PDMS chips and digested with collagenase I (Sigma) at 100 U/ml concentration in Advanced DMEM/F12 with penicillin/streptomycin, 1× Glutamax and 10 mM HEPES, supplemented with 1× B27 supplement (Gibco), 1 µM N-acetylcysteine (Sigma) for 10 min at 37°C with 300 g. Human colon organoids in biological triplicates were collected from the Matrigel domes, and both organoids and mini-colons were dissociated to single cells using 4 mg/mL Protease VIII (Sigma) in dPBS containing 10 µM Y-27632 on ice for approximately 45 min with trituration using a P1000 micropipette every 10 minutes. Following one washing step in PBS+2% BSA (Gibco), cell suspensions were incubated for 20 min on ice with TotalSeq™-C anti-human Hashtag oligos (HTOs) (1:500, Biolegend, 394661, 394663, 394665, 394667, 394669, 394671, 394673, 394675, 394677, 394679, 394683, 394685) in PBS+2% BSA. Cells from each condition and replicate were washed three times with PBS+2% BSA, pooled together and filtered through low-volume 10 µm cell strainers (PluriSelect). All cell suspensions were recounted to achieve a uniform concentration of 2000 cells per microliter before pooling for 10× capture. The cell hashing and cDNA libraries were constructed using 10x Genomics Chromium Next GEM Single Cell 5' Reagent Kits v2 (Dual Index) reagents and sequenced using Illumina protocol using NovaSeq 6000 reagents with around 50000 reads per cell.

IL22-/+ colon organoid

Human colonic epithelial organoids from three healthy individuals were cultured using previously published culture conditions[18]. Organoids were seeded as single cells and treated with IL-22 (10 ng/mL) from day 4 to day 7. Treated and untreated organoids were collected at day 7 and dissociated to single cells using TrypLE (Invitrogen) incubation at 37°C. The six different samples were tagged with different TotalSeqTM

hashtag antibodies (A0251-A0256, Biolegend). Cells were subsequently sorted on an ARIAII FACS sorter (BD) to isolate DAPI negative cells with a previously published gating strategy[19]. A total of 6100 events/cells were isolated from each sample for scRNA-sequencing using the 10X Genomics protocol v3.1 with implementation of hashtag libraries. Additional HTO primer (0.2 μM) was added in the step of cDNA amplification to increase yield of hashtag-barcodes. After the cDNA amplification, hashtag-cDNA and endogenous cDNA were separated based on the size with SPRIselect beads (Beckman Coulter) as described[20]. The dual index kit set AA (10X genomics) were used for sample indexing and 11 cycles were used during the final amplification step. The hashtag cDNA and endogenous cDNA libraries were diluted to 4 nM and pooled (5% hashtag + 95 % endogenous cDNA) before being sequenced on a NextSeq2000 sequencer (Illumina).

References

1. Spence, J. R. *et al.* Directed differentiation of human pluripotent stem cells into intestinal tissue in vitro. *Nature* **470**, 105–109 (2011).
2. Tsai, Y.-H. *et al.* In vitro patterning of pluripotent stem cell-derived intestine recapitulates in vivo human development. *Development* Preprint at https://doi.org/10.1242/dev.138453 (2016).
3. Capeling, M. *et al.* Generation of small intestinal organoids for experimental intestinal physiology. *Methods Cell Biol.* **159**, 143–174 (2020).
4. Heijmans, J. *et al.* ER stress causes rapid loss of intestinal epithelial stemness through activation of the unfolded protein response. *Cell Rep.* **3**, 1128–1139 (2013).
5. Ootani, A. *et al.* Sustained in vitro intestinal epithelial culture within a Wnt-dependent stem cell niche. *Nat. Med.* **15**, 701–706 (2009).
6. Capeling, M. M. *et al.* Suspension culture promotes serosal mesothelial development in human intestinal organoids. *Cell Rep.* **38**, (2022).
7. Holloway, E. M. *et al.* Differentiation of Human Intestinal Organoids with Endogenous Vascular Endothelial Cells. *Dev. Cell* **54**, 516–528.e7 (2020).
8. Watson, C. L. *et al.* An in vivo model of human small intestine using pluripotent stem cells. *Nat. Med.* **20**, 1310–1314 (2014).
9. Finkbeiner, S. R. *et al.* Transcriptome-wide Analysis Reveals Hallmarks of Human Intestine Development and Maturation In Vitro and In Vivo. *Stem Cell Reports* (2015) doi:10.1016/j.stemcr.2015.04.010.

10. Cortez, A. R. *et al.* Transplantation of human intestinal organoids into the mouse mesentery: A more physiologic and anatomic engraftment site. *Surgery* **164**, 643–650 (2018).
11. Gu, W., Colarusso, J. L., Dame, M. K., Spence, J. R. & Zhou, Q. Rapid establishment of human colonic organoid knockout lines. *STAR Protoc* **3**, 101308 (2022).
12. Sato, T. *et al.* Long-term expansion of epithelial organoids from human colon, adenoma, adenocarcinoma, and Barrett's epithelium. *Gastroenterology* **141**, 1762–1772 (2011).
13. Miyoshi, H. & Stappenbeck, T. S. In vitro expansion and genetic modification of gastrointestinal stem cells in spheroid culture. *Nat. Protoc.* **8**, 2471–2482 (2013).
14. Fujii, M. *et al.* Human Intestinal Organoids Maintain Self-Renewal Capacity and Cellular Diversity in Niche-Inspired Culture Condition. *Cell Stem Cell* **23**, 787–793.e6 (2018).
15. Yu, Q. *et al.* Charting human development using a multi-endodermal organ atlas and organoid models. *Cell* **184**, 3281–3298.e22 (2021).
16. Hu, H. *et al.* Long-Term Expansion of Functional Mouse and Human Hepatocytes as 3D Organoids. *Cell* **175**, 1591–1606.e19 (2018).
17. Bartfeld, S. *et al.* In vitro expansion of human gastric epithelial stem cells and their responses to bacterial infection. *Gastroenterology* **148**, 126–136.e6 (2015).
18. Fujii, M., Matano, M., Nanki, K. & Sato, T. Efficient genetic engineering of human intestinal organoids using electroporation. *Nat. Protoc.* **10**, 1474–1485 (2015).
19. JAK/STAT signaling promotes the emergence of unique cell states in ulcerative colitis. *Stem Cell Reports* **19**, 1172–1188 (2024).
20. Stoeckius, M. *et al.* Cell Hashing with barcoded antibodies enables multiplexing and doublet detection for single cell genomics. *Genome Biol.* **19**, 224 (2018).
