# Supplementary material for: An integrated transcriptomic cell atlas of human endoderm-derived organoids
Source: Nat Genet. 2025 May 12;57(5):1201–12. doi: 10.1038/s41588-025-02182-6 (PMC12081310; doi:10.1038/s41588-025-02182-6)
Supplement: Supplementary file 2 — Reporting Summary [file 41588_2025_2182_MOESM2_ESM.pdf]

nature portfolio

Corresponding author(s): Quan Xu
Barbara Treutlein
Fabian J. Theis
J. Gray Camp

Last updated by author(s): Jan 22, 2025

Reporting Summary

Nature Portfolio wishes to improve the reproducibility of the work that we publish. This form provides structure for consistency and transparency in reporting. For further information on Nature Portfolio policies, see our Editorial Policies and the Editorial Policy Checklist.

Statistics

For all statistical analyses, confirm that the following items are present in the figure legend, table legend, main text, or Methods section.

n/a	Confirmed	
☐	☒	The exact sample size (n) for each experimental group/condition, given as a discrete number and unit of measurement
☐	☒	A statement on whether measurements were taken from distinct samples or whether the same sample was measured repeatedly
☐	☒	The statistical test(s) used AND whether they are one- or two-sided<br>*Only common tests should be described solely by name; describe more complex techniques in the Methods section.*
☐	☒	A description of all covariates tested
☐	☒	A description of any assumptions or corrections, such as tests of normality and adjustment for multiple comparisons
☐	☒	A full description of the statistical parameters including central tendency (e.g. means) or other basic estimates (e.g. regression coefficient) AND variation (e.g. standard deviation) or associated estimates of uncertainty (e.g. confidence intervals)
☐	☒	For null hypothesis testing, the test statistic (e.g. F, t, r) with confidence intervals, effect sizes, degrees of freedom and P value noted<br>*Give P values as exact values whenever suitable.*
☒	☐	For Bayesian analysis, information on the choice of priors and Markov chain Monte Carlo settings
☒	☐	For hierarchical and complex designs, identification of the appropriate level for tests and full reporting of outcomes
☐	☒	Estimates of effect sizes (e.g. Cohen's d, Pearson's r), indicating how they were calculated

Our web collection on statistics for biologists contains articles on many of the points above.

Software and code

Policy information about availability of computer code

Data collection	STAR version=2.7.10b
Data analysis	All code used was deposited on Github (https://github.com/devsystemslab/snapseed; https://github.com/devsystemslab/sc2heoca; https://github.com/devsystemslab/HEOCA).<br><br>Versions of main packages used: seq2science (v1.2.2), sc2heoca (v0.3.0), scarches (v0.5.7), scvi-tools (v0.20.3), scib-metrics (v0.3.3), adpbulk (v0.1.3), harmonypy (v0.0.9), bbknn (v1.5.1) scDECAF (v0.99.0), scanpy (v1.9.3)

For manuscripts utilizing custom algorithms or software that are central to the research but not yet described in published literature, software must be made available to editors and reviewers. We strongly encourage code deposition in a community repository (e.g. GitHub). See the Nature Portfolio guidelines for submitting code & software for further information.

Data

Policy information about availability of data

All manuscripts must include a data availability statement. This statement should provide the following information, where applicable:

- Accession codes, unique identifiers, or web links for publicly available datasets
- A description of any restrictions on data availability
- For clinical datasets or third party data, please ensure that the statement adheres to our policy

The HEOCA (raw and normalized counts, integrated embedding, cell type annotations, and technical metadata) is publicly available and can be downloaded via CELLxGENE (https://cellxgene.cziscience.com/collections/6282a908-f162-44a2-99a3-8a942e4271b2) and Zenodo (https://10.5281/zenodo.8181495). The HEOCA core reference model and embedding for the mapping of new data to the HEOCA and human intestinal organoid cell atlas can moreover be found on Zenodo (https://doi.org/10.5281/zenodo.8181495). The GRCh38 genome assembly can be found under (https://www.ncbi.nlm.nih.gov/datasets/genome/GCF_000001405.26/). The scRNA-seq data of intestine organoid generated in this study have been deposited in the Gene Expression Omnibus (GEO) database under the accession number GSE287233

Research involving human participants, their data, or biological material

Policy information about studies with human participants or human data. See also policy information about sex, gender (identity/presentation), and sexual orientation and race, ethnicity and racism.

Reporting on sex and gender	N/A
Reporting on race, ethnicity, or other socially relevant groupings	N/A
Population characteristics	N/A
Recruitment	N/A
Ethics oversight	N/A

Note that full information on the approval of the study protocol must also be provided in the manuscript.

Field-specific reporting

Please select the one below that is the best fit for your research. If you are not sure, read the appropriate sections before making your selection.

☒ Life sciences ☐ Behavioural & social sciences ☐ Ecological, evolutionary & environmental sciences

For a reference copy of the document with all sections, see nature.com/documents/nr-reporting-summary-flat.pdf

Life sciences study design

All studies must disclose on these points even when the disclosure is negative.

Sample size	We collected all the representative scRNA-seq data sets of different human endoderm-derived organoids protocols that are accessible
Data exclusions	Quality control was applied to exclude cells with low quality. Detailed methods are described in the Methods section (page 38)
Replication	For the mini colon-organoids, replicates were successfully performed as indicated in this previously published paper (https://www.cell.com/cell-stem-cell/fulltext/S1934-5909(24)00184-X). Iindividual replicates demonstrated a high degree of uniformity, highlighting the consistency of the experimental procedures and reproducibility of the system. For the atlas we integrated the previously published samples into one embedding to study cross-organoid-protocol overlap and reproducibility. For our generated differentiation and perturbation protocols we used two separate successful controls for the experiments.
Randomization	This study is mainly an accumulative effort of existing published data for which a classical blinding strategy does not apply in our opinion. For integration method validation and the factor effect integration validation, we randomly selected samples. Generally, all compared or combined samples were integrated and analyzed with the same computational parameters and strategies. For integration method validation and the factor effect integration validation, we randomly selected samples.
Blinding	This study is mainly an accumulative effort of existing published data for which a classical blinding strategy does not apply in our opinion. All combined samples were integrated and analyzed with the same computational parameters and strategies to ensure comparability.

Reporting for specific materials, systems and methods

We require information from authors about some types of materials, experimental systems and methods used in many studies. Here, indicate whether each material, system or method listed is relevant to your study. If you are not sure if a list item applies to your research, read the appropriate section before selecting a response.

Materials & experimental systems

n/a	Involved in the study
☐	☒ Antibodies
☐	☒ Eukaryotic cell lines
☒	☐ Palaeontology and archaeology
☐	☒ Animals and other organisms
☒	☐ Clinical data
☒	☐ Dual use research of concern
☒	☐ Plants

Methods

n/a	Involved in the study
☒	☐ ChIP-seq
☒	☐ Flow cytometry
☒	☐ MRI-based neuroimaging

Antibodies

Antibodies used

TotalSeq™-C anti-human Hashtag oligos (HTOs) (1:500, Biolegend, 394661, 394663, 394665, 394667, 394669, 394671, 394673, 394675, 394677, 394679, 394683, 394685); TotalSeqTM hashtag antibodies (A0251-A0256, Biolegend) were used according to manufacturer's instructions (0.5 mg per sample, https://doi.org/10.1016/j.stemcr.2024.06.006).

Validation

Each lot of this antibody is quality control tested by immunofluorescent staining with flow cytometric analysis and the oligomer sequence is confirmed by sequencing. TotalSeq™-C antibodies are compatible with 10x Genomics Chromium Single Cell Immune Profiling Solution.

Relevant citations provided by the manufacturer:

TotalSeq™-C anti-human Hashtag oligos (HTOs) (Biolegend, 394661):

Liu C, et al. 2021. Cell. 184(7):1836-1857.e22. PubMed
Li SS, et al. 2022. Cell Host Microbe. 30:1173. PubMed
Liu Y, et al. 2023. Nat Commun. 14:2179. PubMed
Collora JA, et al. 2023. Genome Res. . PubMed
Sudmeier LJ, et al. 2022. Cell Rep Med. 3:100620. PubMed
Yu B, et al. 2022. Cell. 185:4904. PubMed
Chow A 2023. Immunity. 56(1):93-106.e6. PubMed
Collora JA, et al. 2022. Immunity. 55:1013. PubMed
Witkowski M, et al. 2021. Nature. 600:295. PubMed
Wagner KI, et al. 2022. Cell Rep. 38:110214. PubMed
Sen K, et al. 2021. Front Immunol. 12:733539. PubMed
Shangguan S, et al. 2021. Elife. 10:. PubMed

TotalSeqTM hashtag antibodies (A0251, Biolegend):

Lombardi O, et al. 2022. Cell Rep. 41:111652. PubMed
Tamaoki N, et al. 2023. Cell Rep Methods. 3:100460. PubMed
Law AMK, et al. 2022. Adv Sci (Weinh). 9:e2103332. PubMed
Meyer M, et al. 2020. Cell Syst. 0.713194444. PubMed
Kaufmann M, et al. 2021. Med. 2(3):296-312.e8. PubMed
Stuart T, et al. 2019. Cell. 177:1888. PubMed
Sui L, et al. 2021. JCI Insight. 6:e141553. PubMed
Benjamin Krämer, et al. 2021. Immunity.. Online ahead of print. PubMed
Witkowski MT, et al. 2020. Cancer Cell. 37:867. PubMed
Nadeu F, et al. 2022. Nat Med. 28:1662. PubMed
Still C 2nd, et al. 2021. Cell Reports Medicine. 2(7):100343. PubMed
Yao C, et al. 2020. Cell Reports. 34(1):108590. PubMed

Eukaryotic cell lines

Policy information about cell lines and Sex and Gender in Research

Cell line source(s)

Time course: HUB-02-A2-040, HUB-04-A2-001, HUB-HS-02-A2-M21-00050, HUB-HS-02-A2-M21-00225, HUB-HS-02-A2-M21-00081, HUB-HS-02-A2-M21-00164, HUB-HS-02-A2-M21-00244, HUB-HS-02-A2-M21-00258, HUB-HS-02-A2-M21-00271, HUB-HS-02-A2-M21-00047. Tissue material was originally obtained from patients included in HUB-Cancer protocol (12- 093). Additional details can be found under this Preprint: https://www.biorxiv.org/content/10.1101/2023.12.18.572103v1.full
Transplanted intestinal organoids: iPSC72.3, H9
ASC organoids for differentiation and perturbation experiments were derived from the healthy ileum tissue of a 50-year old female who underwent resection of a malignant tumor of the colon ascendens.

Authentication

ASC organoids for differentiation and perturbation experiments were derived from the healthy ileum tissue of a 50-year old

Authentication	female. The untransformed status of the origin tissue was confirmed by a pathologist.
Mycoplasma contamination	All ASC cultures used in the differentiation and perturbation experiments were tested negative for mycoplasma.
Commonly misidentified lines (See ICLAC register)	-

Animals and other research organisms

Policy information about studies involving animals; ARRIVE guidelines recommended for reporting animal research, and Sex and Gender in Research

Laboratory animals	Immunocompromised NOD-SCID IL2Rg null (NSG) mice (strain no. 0005557) were used in organoid transplantation experiments.<br><br>In accordance with the guidelines for facilities, housing, and environmental management set forth by the Guide for the Care & Use of Laboratory Animals, the University of Michigan Unit for Laboratory Animal Medicine (ULAM) uses an established set of standard lighting practices in all animal housing rooms on campus. Housing rooms employ centrally controlled and monitored light cycles that utilize a 12-hour light / 12-hour dark photoperiod. Temperatures are maintained within plus or minus 2 degrees throughout a range of ~18-26°C with 30-70% humidity.
Wild animals	We do not use wild animals in this study.
Reporting on sex	Mice were solely used for transplantation experiments for human organoids and we therefore did not analyze any potentially male or female biased mouse gene expression data of either sex in this study.
Field-collected samples	We do not use field-collected samples in this study.
Ethics oversight	Institutional Animal Care and Use Committee (Protocol # PRO00006609)

Note that full information on the approval of the study protocol must also be provided in the manuscript.

Plants

Seed stocks	*Report on the source of all seed stocks or other plant material used. If applicable, state the seed stock centre and catalogue number. If plant specimens were collected from the field, describe the collection location, date and sampling procedures.*
Novel plant genotypes	*Describe the methods by which all novel plant genotypes were produced. This includes those generated by transgenic approaches, gene editing, chemical/radiation-based mutagenesis and hybridization. For transgenic lines, describe the transformation method, the number of independent lines analyzed and the generation upon which experiments were performed. For gene-edited lines, describe the editor used, the endogenous sequence targeted for editing, the targeting guide RNA sequence (if applicable) and how the editor was applied.*
Authentication	*Describe any authentication procedures for each seed stock used or novel genotype generated. Describe any experiments used to assess the effect of a mutation and, where applicable, how potential secondary effects (e.g. second site T-DNA insertions, mosiacism, off-target gene editing) were examined.*
